# What is the effectiveness of services and interventions for Armed Forces transitioning to veteran status, and what opportunities are there for improvement: a scoping review of health-related evidence

**DOI:** 10.1136/bmjopen-2026-122999

**Published:** 2026-09-29

**Authors:** Jessica M Armitage, Sophie Robinson, Jamie Bowater, G J Melendez-Torres, Maxwell S Barnish

**Affiliations:** 1 Public Health and Sport Sciences Medical School, University of Exeter, Exeter, UK; 2 Centre for Public Health and Wellbeing, School of Health and Social Wellbeing, University of the West of England, Bristol, UK

**Keywords:** Public health, MENTAL HEALTH, Review

## Abstract

**Abstract:**

**Objectives:**

To identify and map health-related services and interventions that support Armed Forces transitioning to veteran status, and to synthesise evidence on their potential effectiveness.

**Design:**

Scoping review.

**Data sources:**

Medline (Ovid), Embase (Ovid), APA PsycInfo (Ovid), Cochrane (Wiley), Web of Science (Clarivate), ASSIA (ProQuest) and PTSD Pubs (Proquest), citation chasing and web searches.

**Participants:**

Armed Forces members, veterans, family members and service providers from the UK, Europe, Canada, Australia or New Zealand.

**Interventions:**

Health-related services and interventions intended to support military to civilian transition.

**Primary and secondary outcome measures:**

Health and wellbeing outcomes (eg, mental health symptoms, quality of life), broader transition-related outcomes (eg, employment, housing, social connection), acceptability, barriers and facilitators to intervention or service engagement.

**Analytical methods:**

Tabulation and narrative synthesis (using thematic analysis for qualitative studies).

**Results:**

Thirty-four unique studies were included, reporting data on 10 040 veterans and transitioning service personnel. Twenty-two distinct interventions were identified and grouped into five categories: (1) art-based, (2) sport or outdoor-based, (3) peer- or group-based, (4) employment, skills or education training and (5) digital technology interventions. Most evidence originated from the UK, with additional studies from Canada, Australia, New Zealand and Israel. While no critical appraisal of study quality was performed within this review, quantitative evaluations suggested benefits for mental health, wellbeing and reintegration outcomes for some intervention types; however, evidence was heterogeneous and variable in outcome measurement. Qualitative evidence highlighted value in social connection, identity and purpose, and identified barriers related to awareness, access, acceptability and the need for tailored provision.

**Conclusions:**

A diverse range of services and interventions are available to support transition to civilian life, but the strength and consistency of the evidence varied considerably, meaning findings should be interpreted as identifying potentially valuable approaches rather than established best practices. Future research should prioritise robust evaluation designs, consistent outcome reporting and attention to implementation and equity. Service planners may benefit from improved signposting and better integration across health, social care and third-sector pathways to support veterans during transition.

**Study registration:**

Zenodo record 17630661.

**STRENGTHS AND LIMITATIONS OF THIS STUDY**

- A broad inclusion strategy that integrated quantitative, qualitative and mixed-methods evidence was used.
- A thorough literature search was conducted using established methodological guidance, relevant grey literature, duplicate screening and stakeholders.
- This was a scoping review therefore no risk-of-bias appraisal was undertaken.
- The evidence base was highly heterogeneous, limiting the ability to draw firm conclusions about comparative effectiveness or generalisability.
- Restriction to studies published in the English language may have resulted in the omission of relevant evidence.

## Introduction

 The transition from military to civilian life presents substantial challenges for veterans, including navigating complex healthcare systems, accessing financial assistance, securing employment and housing, as well as rebuilding connections and identity.^1 2^ For some, changes to identity, culture and routine can cause great difficulty during transition, and a loss of social networks can increase feelings of loneliness and present barriers to help seeking.^3 4^ These problems may predict or coincide with poorer mental health and wellbeing, posing far-reaching consequences for both the veteran and their family.^3 4^

Common mental health disorders are prevalent among veterans. In 2022–2023, up to 28% of veterans in the UK reported experiencing such difficulties, an increase of nearly 8% compared with surveys carried out in 2004–2006.^5^ The prevalence of common mental health disorders is generally higher than those observed in civilian populations,^6^ with veterans at a further increased risk of post-traumatic stress disorder (PTSD) and alcohol misuse.^5 7^ Rates of alcohol misuse are particularly pronounced, with estimates around two to three times higher than those in the general population.^5^ Given these multifaceted challenges associated with reintegration, understanding the availability and effectiveness of interventions for veterans is essential to supporting their transition and promoting long-term health and wellbeing.

Since 2001, the landscape of veteran support has changed substantially, with both governmental and non-governmental organisations expanding the range and scope of services and interventions available to transitioning service members.^8^ Many of these involve strategies designed to tackle both psychological and social factors associated with transition.^9^ These interventions have taken the form of physical activity or outdoor-based courses,^10^ peer support groups,^11^ employment and vocational-based interventions,^12^ digital interventions,^13^ as well as creative practices.^14 15^ While reviews of the evidence point towards some positive benefits, many often focus on one outcome or one intervention type.^16^ Few have integrated findings across interventions targeting physical, psychological and social outcomes for veterans, and those that do focus on evidence from the USA.^8^ The USA has a distinct veteran support landscape which involves the Department of Veterans Affairs, and differing eligibility pathways for benefits and healthcare. This is likely to impact military culture and experiences both during and post-service,^17^ meaning findings from US studies may not be directly transferable to other contexts. Given that existing reviews are heavily dominated by US evidence, an important gap remains in synthesising evidence from settings with comparable systems of veteran support and transition.

Veteran support has evolved over time in response to changing veteran needs, meaning there is a corresponding need to evaluate the effectiveness of contemporary interventions and identify areas for further service development. The aims of the present review were therefore to identify and map health-related services and interventions that support Armed Forces members transitioning to veteran status in European countries and non-US Five Eyes Alliance nations (United Kingdom, Canada, Australia and New Zealand). Specifically, we aimed to summarise the range of available provision, synthesise evidence on possible effectiveness across physical, psychological and social domains, as well as highlight opportunities for future service development and improvement. We applied a mixed methods approach to evidence synthesis and integrated both quantitative and qualitative evidence. The aim was to provide a more holistic account of what works, for who, and in what contexts, enabling consideration of outcomes alongside contextual factors.

## Methods

### Design

A scoping review was conducted following the Joanna Briggs Institute^18^ and Preferred Reporting Items for Systematic Reviews and Meta-Analyses—Scoping Reviews (PRISMA-ScR)^19^ and registered on the Zenodo database (record 17630661). The PRISMA-ScR^19^ checklist is provided as an online supplemental file 1, and the PRISMA flow diagram can be found in figure 1.

**Figure 1:**
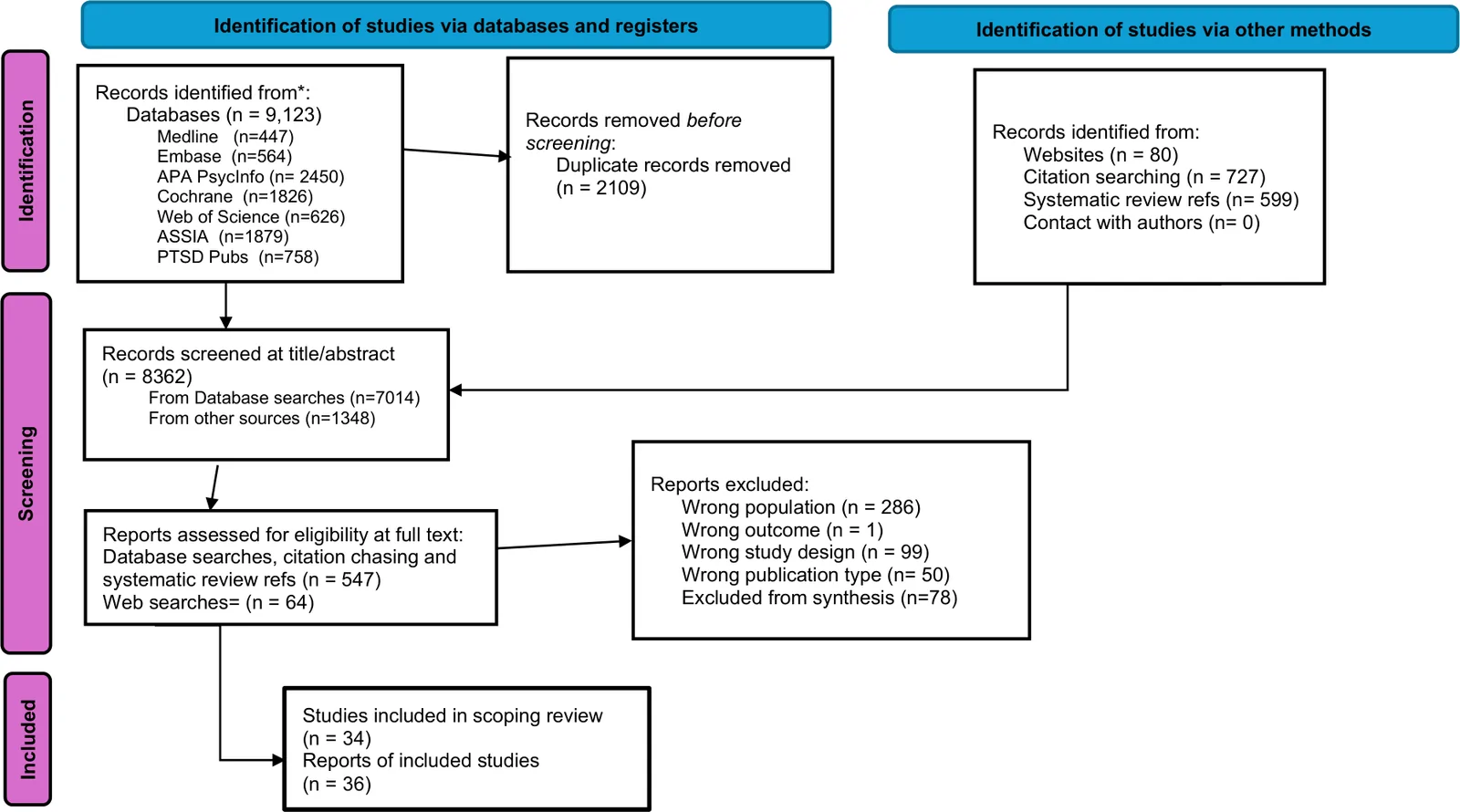
Preferred Reporting Items for Systematic Reviews and Meta-Analyses (PRISMA) flow diagram. Flow diagram showing the identification, screening, eligibility assessment and inclusion of studies in the review. Source: Page MJ, *et al*. BMJ 2021;372:n71. doi: 10.1136/bmj.n71. This work is licensed under CC BY 4.0. To view a copy of this license, visit https://creativecommons.org/licenses/by/4.0/.

### Literature search strategy

Searches were undertaken in November 2025 across seven databases: Medline (Ovid), Embase (Ovid), APA PsycINFO (Ovid), Cochrane (Wiley), Web of Science (Clarivate), ASSIA (ProQuest) and PTSD Pubs (ProQuest). The search strategy was part of a broader project comprising two scoping reviews as part of the James Lind Alliance Priority 5. The search strategy included terms related to veterans, discharge from service and terms for the Five Eyes nations (minus USA) and Europe. Supplementary literature searching involved checking reference lists of systematic reviews, citation chasing and web searches to identify grey literature and policy documents. All searches were designed and conducted by an information specialist (SR). Full search strategies, including web searches, are shown in online supplemental file 2.

### Eligibility criteria

We included veterans who had served for at least 1 day in the Armed Forces (Regular or Reserve) or merchant mariners, and seen duty on legally defined military operations. Veterans were of any age from the UK, Europe, Canada, Australia or New Zealand. The WHO definition of the European Region was used to define Europe. Veterans at the point of transition from current military service to veteran status were included, as were those providing data relevant to the period of transition. Studies where the participants were service providers, relatives or friends, rather than veterans themselves, were included if the focus of the research and outcomes related to veterans. Eligible services or interventions were those aimed at improving the health or wellbeing of individuals transitioning from military service to veteran status. The potential effectiveness of interventions or services was determined by including those with comparative measures assessed in an earlier time period, as well as those where individuals underwent no intervention or service comparison. Comparisons across current service provision were also included. Studies unrelated to effectiveness did not require a comparator.

While a formal quality appraisal was not conducted, intervention or service effectiveness were determined using health and wellbeing outcomes, including patient- and clinician-reported outcomes, diagnostic or symptom tests, as well as broader wellbeing outcomes, including housing, employment and education. Eligible study designs included qualitative studies, quantitative studies, including observational cross-sectional or cohort studies, randomised controlled trials (RCTs) and mixed method designs. Relevant systematic or scoping reviews were used to identify studies and were then excluded.

We considered studies published in the English language only, and included peer reviewed papers, theses, dissertations and official reports. Grey literature was included using the same eligibility criteria, data extraction procedures and synthesis approach as peer-reviewed evidence. Conference abstracts were excluded due to a lack of data.

### Screening and data extraction

Screening was initially conducted based on the title and abstract. Potentially relevant articles were screened at the full-text stage to determine inclusion or exclusion. All records were screened independently by three reviewers (JMA, MSB and JB) and disagreements resolved through discussion among all three reviewers. All screening was conducted using Rayyan.

Three reviewers conducted data extraction (JMA, MSB and JB). All records were extracted by one reviewer and independently checked by a second reviewer. A predefined data extraction framework was used and disagreements were resolved through discussion among all three reviewers. The following information was extracted from each included study: the authors, year of publication, country in which the research was conducted, including any geographical limitations, year(s) of data collection, the study design and the recruitment route. Key characteristics such as sample size, population and inclusion and exclusion criteria were also recorded. Information on the interventions and comparators were also extracted, as well as primary and secondary outcomes assessed. Data on analysis methods, funding sources and declared conflicts of interest were collected to support assessment of methodological quality and potential biases. Study results were extracted and reported separately for quantitative and qualitative data to ensure comprehensive synthesis across diverse methodological approaches. No automation tools were used during screening or data extraction.

### Data synthesis

Following data extraction, tables were produced to characterise study designs (online supplemental file 3), veteran profiles (online supplemental file 4), quantitative results (online supplemental file 5) and qualitative results (online supplemental file 6). Data were subsequently analysed using narrative synthesis, exploring outcomes related to the potential effectiveness of interventions and opportunities for improvement. Data synthesis was first conducted separately for quantitative and qualitative evidence, with thematic analysis used to synthesise qualitative data. An integrated synthesis of all evidence was then carried out and an evidence map created (figure 2). Intervention types were identified and categorised inductively during data synthesis based on their primary aims and characteristics. Any uncertainties or disagreements regarding categorisation were resolved through discussion and consensus.

**Figure 2:**
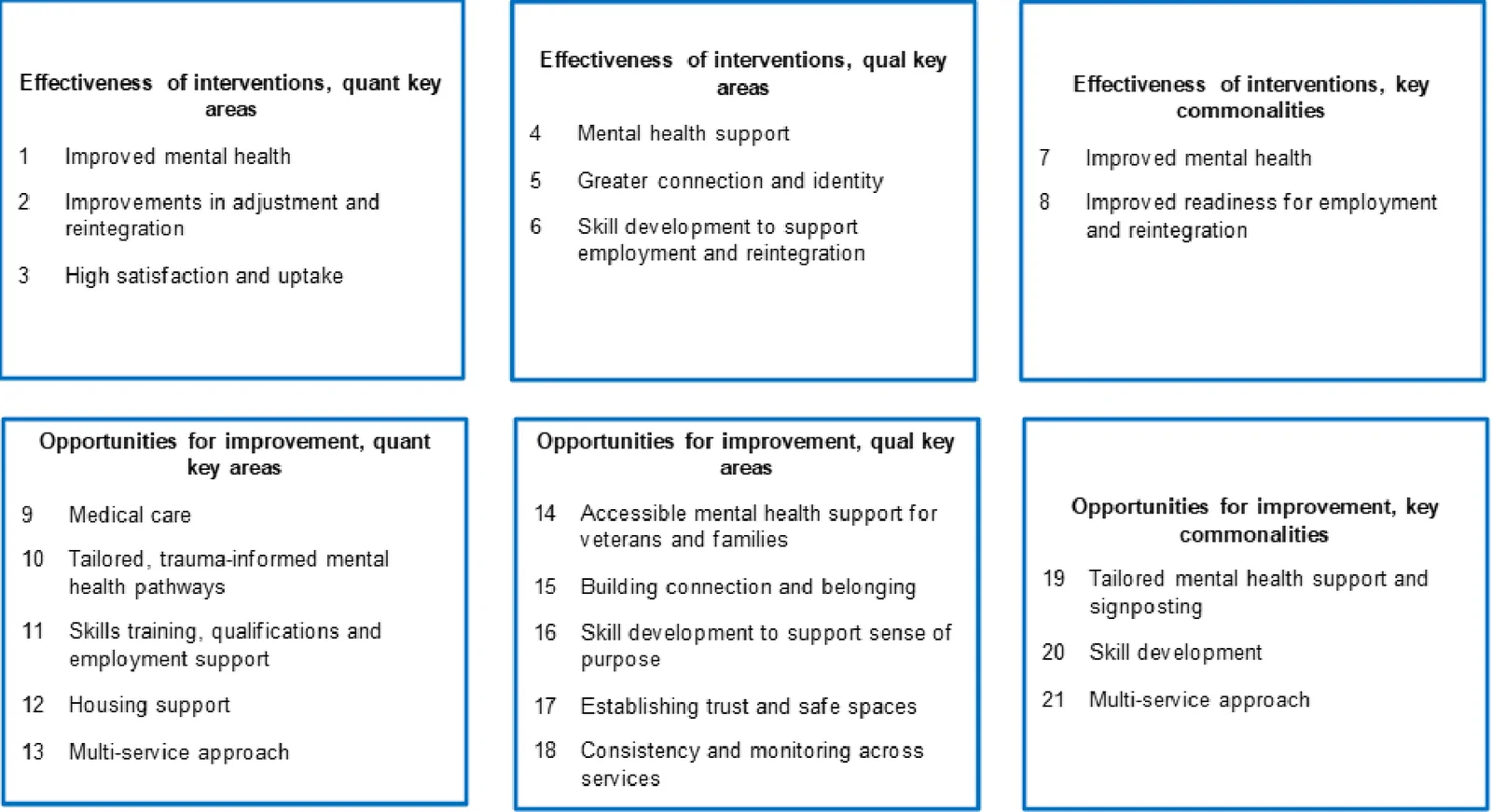
Evidence map. Evidence map summarising qualitative and quantitative evidence across identified themes.

### Patient and public involvement and engagement

Stakeholders across the Ministry of Defence (UK), NHS England, and the Department for Health and Social Care were involved in the generation of the topic brief and were provided the opportunity to comment on a draft report.

## Results

The database search yielded 7014 citations, with a further 1268 identified from supplementary searches. Screening of titles and abstracts resulted in 611 records for full-text review, of which 36 were ultimately included, representing 34 unique studies. Online supplemental file 7 contains a list of included studies.

### Study profiles

Across the included studies, information relating to 10 040 veterans and transitioning military personnel was available, with sample sizes ranging from 6 to 5399. Of these, 30 involved assessments with veterans, 8 with service providers, 4 with military service members preparing for discharge and 2 with family members. For studies conducted solely on veteran populations, time since discharge was not typically stated or was variable and no studies looked specifically at those recently discharged.

A wide age range of veterans was included, with just one study^20^ focusing on young service leavers. Several studies involved both male and female veterans, with seven^12 21–26^ focused on only male participants, and three^27–32^ on females only. Nearly two-thirds of studies included participants based in the UK, with one also including veterans who served in the French Foreign Legion.^33^ One further study^34^ included UK veterans, alongside those from Australia, Canada and the USA. Among the remaining studies, five^10 21 22 25 35^ were Canadian, four^36–39^ were Australian, one^20^ was Israeli, one^40^ was based in New Zealand. Studies included veterans from a variety of branches of the Armed Forces, although most participants were from the Army. There were no studies specific to the Navy or Air Force. Additional information related to the characteristics of veteran and transitioning military service personnel can be found in online supplemental file 4.

### Narrative synthesis

Of the 34 unique records, 24 provided quantitative or qualitative data related to specific interventions, services or charities, with others providing evaluations of multiple services. In other reports, two described the effectiveness of services provided by specific organisations or charities, including the Veterans Advocacy People (Forces in Mind, 2021)^41^ and Salute Her,^30^ while other studies described multiple services. One study documented the codesign of a workshop aimed at reducing problematic alcohol use among veterans transitioning.^40^ See table 1 for an overview of the main approaches, and full details in online supplemental file 8. Figure 2 presents an evidence map demonstrating key areas of convergence between the quantitative and qualitative evidence.

**Table 1:** Overview of intervention types

Intervention type	Overview	Studies
Art-based	Art-based interventions involved using structured arts and performance programmes to support veterans’ recovery, identity rebuilding and wellbeing. Most initiatives used professional theatre practitioners and actors to support veterans in safely sharing their story through language, action and emotion,^22 23^ with psychological support provided throughout.^33^ Some interventions involved family members to explore experiences of coming home	Ackley and Wilson-Menzfeld^33^; Belliveau *et al*^22 23^; O’Connor^24 42^
Sport or outdoor-based	Sport-based initiatives include the Outward Bound Experience,^10^ Invictus Games,^43^ the Purpose After Service through Sport (PASS) programme,^25^ surf therapy,^36^ the UK Sport for Development (SFD) model,^44^ as well as military-paralympic programmes.^35^ All use physical activity as a foundation for rehabilitation, social connection and reintegration for veterans. Some, including the surf therapy and PASS, involve weekly sessions open to all veterans with the aim of combining goal setting, sports practice and informal social interactions, while others, like the Outward Bound Experience, involve 7-day expeditions.	Brittain *et al*, 2012^34^; Harper *et al*^10^; Mackintosh *et al*^43^; Moran *et al*^36^; O’Hanlon *et al*^44^; Waldhauser *et al*^25^
Group-based	Group-based programmes address a range of emotional, interpersonal and career-related barriers that may impact successful transition. Approaches often involve psychoeducation, emotion regulation and cognitive-behavioural therapy techniques to manage mental health difficulties, as well as therapeutic enactment.^21 35 39^ The focus of many group-based programmes is on developing a safe space wherein veterans can experience mutual support and social connections.^39 45^ Many also involve skills training and goal setting to equip veterans with a clear plan for transitioning into civilian life^20 21 35 39^	Albertson^45^; Cox *et al*^35^; Gati *et al*^20^; Romaniuk *et al*^39^; Westwood *et al*^21^
Employment or skills training	Three initiatives supporting veterans’ transition to civilian life through employment or skills-based training were included. Two of these included dedicated employment training programmes or courses, including one provided by the Scotland’s Bravest Manufacturing Company (SBMC)^12^ and another from Building Heroes.^46^ The other was a pilot randomised controlled trial testing attention control training.^37^	Hutcheon *et al*^12^; Meierdirk^46^; Metcalf *et al*^37^
Digital interventions	Two digital applications were evaluated in the UK, the Mental Health Toolkit for Veterans (MeT4VeT)^27^ and Drinks:Ration.^47^ The MeT4VeT aimed to support UK Armed Forces veterans experiencing mental health difficulties, while Drinks:Ration focused on reducing harmful alcohol consumption. Outside of the UK, the ‘Go-Beyond,^38^ Navigating Life Beyond Service’ web-based programme was developed to provide links to education, services and support in Australia.	Alichniewicz *et al*^38^; Parkes *et al*^27^; Williamson *et al*^47^

### The effectiveness of different interventions for veteran transition

Out of the studies that included an intervention, five used an arts-based theatre approach,^22–24 33 42^ six involved a sports or outdoor-based intervention,^10 25 34 35 43 44^ five used peer- or group-based interventions to deliver support,^20 21 35 39 45^ three involved employment, skills or education training,^12 37 46^ and three were digital technology interventions^27 46 47^ (see table 1). The intervention categories were developed through an iterative process of data extraction and thematic grouping. Following identification of studies that included an intervention, key characteristics of each intervention (eg, design, primary activity, analysis and outcomes) were extracted and compared across studies. All art-based interventions used qualitative methods for data collection, while all digital technology interventions used quantitative data. Those using sport, outdoor-based, group-based and employment, skills or education training used a combination of the two.

### Art-based approaches

Qualitative research revealed that theatre-based interventions aimed at supporting veterans can improve wellbeing through improving connection, sense of purpose, confidence and trust.^24 42^ In the UK, the Coming Home programme helped to reconnect veterans and their families with their communities, with the emotional release afforded by singing and performing together described as particularly powerful.^42^ Participants said that the programme helped them reconnect with aspects of themselves they thought had been lost, including humour, spontaneity and fun, which they associated with improvements to their wellbeing. Some described the arts-based approach as not being therapy ‘but is certainly therapeutic’.^24^

### Sport or outdoor-based approaches

Similar enhancements to wellbeing were noted through sport or outdoor-based approaches, including the Purpose After Service through Sport programme in Canada.^25^ Through weekly games of ball hockey and opportunities to socially connect afterwards, veterans reported benefits to both mental and physical health, as well as social connections and access to resources.^25^ Similar positive results were obtained from a non-randomised control study in Australia following a surf therapy intervention programme.^36^ The study was the largest quantitative surf therapy study and revealed significantly reduced symptoms of PTSD (β=−11.92, 95% CI −17.44 to 6.36), depression (β=−7.87, 95% CI −12.35 to 3.38) and anxiety (β=−4.02, 95% CI −6.42 to −1.57), with all changes retained at 7 months follow-up. No changes were observed in the control group.

Other outcomes relevant to wellbeing that also improved through sport or outdoor-based activities include self-confidence, resilience, connection and empowerment, with medium to large effect sizes noted 6 weeks following completion of the Outward Bound Canada course.^10^ Interviews with veterans completing the programme noted that they found meaning through intense group dialogue and physical activity.

### Group-based approaches

Group-based approaches that do not involve sport have shown mixed effects on mental health outcomes. In Canada, participation in a Veterans Transition Program led to improvements in trauma symptoms, depression, self-esteem and wellbeing immediately and at 3-month follow-up.^21^ In contrast, an Australian evaluation of a group-based intervention grounded in cognitive–behavioural and acceptance and commitment therapy techniques found no significant changes in quality of life, depression, anxiety or stress following 8 weekly sessions.^39^ Further evidence from a different transition programme in Australia suggested that any benefits of a multimodal transition programme may be inhibited by existing PTSD symptoms.^35^

Similarly, mixed results emerge for group-based approaches when considering employment outcomes. In Australia, the study focused on therapy techniques revealed that despite no improvements to mental health or wellbeing, veterans reported improvements in general adjustment, reflected in a marked improvement in M-CARM (Military–Civilian Adjustment and Reintegration Measure) scores from preintervention to postintervention.^36^ In Israel, a 5-day workshop revealed mixed effects on career decision-making difficulties and beliefs by the end of the workshop.^20^

### Employment and skills training

Employment outcomes appear more consistently positive following dedicated employment or skills training programmes. Qualitative evidence from Scotland’s Bravest Manufacturing Company, which aims to prepare veterans for employment, showed a greater sense of confidence in being work-ready,^12^ with many reporting a greater sense of pride and self-worth.^12^

Skills training in other areas, such as attention-control, can also improve outcomes for veterans. In a RCT in Australia, four sessions of attention-control training resulted in significantly lower levels of PTSD and improved work and social adjustment.^37^

### Digital technology

Digital interventions have proven particularly effective in improving veterans’ mental health in the UK. Quantitative research has shown that a Mental Health Toolkit resulted in significant reductions to mental health distress at 3 months follow-up, with general health scores (GHQ-12) improving by a mean of 4.3 points (95% CI 1.9 to 6.7), and PTSD scores (PCL-C) decreasing by 11.0 points (95% CI 1.3 to 20.7).^27^ Wellbeing and quality of life also improved from preintervention to postintervention. No differences in any of the outcome measures were noted for the control group.^27^ Quantitative findings from another smartphone-based intervention reported similar improvements in psychological wellbeing and physical health, with benefits also noted for substance use.^47^

### Individual charities or organisations

Evidence from individual organisations suggested that veterans who engaged with the Veterans Advocacy People in the UK experience improvements in health and mental wellbeing, with many emphasising that the service enabled timely access to healthcare before problems escalated to crisis.^41^ Many went on to also access education or secure a job, while others had their debt either cleared or made subject to a new and manageable payment agreement.^41^ Several were also assisted in resolving housing issues, accessing a mobility vehicle, as well as tackling financial and benefit issues.^41^

Greater quality of life was also reported among female veterans engaged with the Team Salute Her UK.^30^ Several described feeling safe, not judged and listened to for the first time since being in military.

### Opportunities for improvement

Evidence from both quantitative and qualitative studies was used to explore opportunities to improve services supporting veterans’ transition.

### Medical care and health needs

Veterans experiencing limitations in daily activities are markedly more likely to prioritise medical care and support for improving transition compared with those without long-term physical or mental health conditions (29.1% vs 6.2%).^3^ This underscores the importance of early identification of physical health needs and tailored signposting to medical services.

### Tailored and inclusive mental health support

Greater emphasis is needed on the emotional and psychological challenges associated with transition.^39 48^ Support should extend to families, helping them to prepare for what to expect during transition and where they can go for advice. This requires accessible information delivered in clear language and via well-marketed websites and social media.^48^ Digital support for veterans should likewise use simple, non-judgemental, engaging and suitable for a range of technical abilities.^47^

Mental health and wellbeing initiatives should adopt a holistic approach rather than target isolated issues such as alcohol abuse,^40^ and veteran-specific mental health services should be responsive to the gendered nature of care needs and the preferences of women veterans.^28 31 49^ Demand for mental health support varies according to gender and service experiences, with females and individuals subject to bullying, discrimination or harassment during service more likely to rate mental health support as important in improving their transition.^3^ Female-friendly services are crucial, but services need to work together to meet the needs of women veterans.^31^

### Improving signposting, identification and military awareness

A key area flagged for improvement was on better signposting of veteran support and healthcare services.^3 41 46 50 51^ According to included reports from the UK, many general practitioners (GPs) practices are not aware of the services and support available to veterans, and do not routinely offer targeted signposting or referrals.^51^ Veterans note that they prefer to seek help from charities and local veteran support groups as they have a much better understanding of their needs.

A key barrier to access is the inconsistent identification of veterans within primary care, many noted that GP practices do not record or identify veterans, meaning there is an onus on veterans to disclose their military service.^51^ Veterans are often reluctant or not aware of the importance of sharing their military history, meaning access to services can be delayed or prevented. Systematic recording of veteran status in GP practices is essential to ensuring timely access to treatment and appropriate care.

Effective communication of available services also requires improvement. Information is often delivered as part of a long list of charities and websites, and there is limited support to navigate these lists or find the relevant organisations.^41^ Veterans therefore report a preference for resettlement information to be communicated and managed one-to-one.^41^ Tailored signposting may be particularly important for female veterans, who often have difficulty identifying as a veteran, and therefore may not consider services relevant to them.^28^ Ensuring services are relevant and show visibility of female veterans in imagery can make it clearer that services are not just for men or combat-related trauma.^28^

Improving service provider military knowledge and understanding of female veterans’ needs is also seen as critical.^28 32 50^ Veterans have suggested that awareness could help reduce the stigma and misconceptions caused by a lack of understanding and recognition of women’s experiences in the military. The use of female veterans as experts-by-experience has been proposed as a mechanism to support the development of service design and delivery.^50^

### Building trust, safe spaces and a sense of belonging

A consistent finding across services was the importance of trust and psychologically safe environments in supporting positive transition outcomes. Veterans have emphasised the importance of trust as they can be particularly vulnerable during the transition^40^ and noted the importance of support and encouragement of the training manager.^12^ Perceptions of facilitators as being warm, caring people who showed openness and genuineness was identified as the second most helpful factor from the group-based Veterans Transition Program in Canada.^21^

For female veterans, building trust was particularly important, with several highlighting the need for healthcare professionals to be aware of female veteran needs in gendered areas of care, such as the perinatal period, menopause and infertility.^32^ Female-specific initiatives and service providers have been seen as valuable for creating a ‘safe space’ where female veterans can discuss gender-specific issues.^32^ However, opinions are divided as to whether these need to be run solely by women. Some felt that there was no need as females identified more with their military role, emphasising the importance of connecting with services as veterans rather than as women.^32^ Others argued for a need for female-specific services because existing services are designed for and by men, and do not address the unique needs and experiences of female veterans.

In addition to establishing trust, the importance of creating and maintaining a shared sense of belonging was also emphasised. A renewed sense of belonging through group sessions encouraged collective reflection and sharing,^39^ and this is seen as particularly effective in helping to build connection,^45^ often reminiscent of their military social ties.^33^ During the Veterans Transition Program in Canada, many identified being with other military personnel as the single most important factor for their experience of change,^21^ with others emphasising the importance of continued connection to Armed Forces^3^ and social connection in supporting postservice life.^40^ During the Outward Bound Experience in Canada, many found meaning through group dialogue, and left the course with an increased understanding of their common ‘soldiers experience’.^10^ Participants acknowledged the value of ‘rank’ not being disclosed to ensure equality and increased cohesion. For female veterans, the sense of belonging and camaraderie provided by services was key to facilitating access to support.^32^

### Creating purpose

A key attribute to the success of some of the programmes was their ability to create a sense of purpose for veterans. Nearly half (46.7%) of veterans said training or additional training would have improved their transition to civilian life, with particularly high value placed on learning new skills or trades (37.1%), gaining qualifications (31.7%) and confirmed employment (32.9%).^3^ For others, engaging in theatrical programmes provided a sense of purpose in terms of routine, skill development and pride in participation.^33^ For others, engaging in sport allowed them to negotiate their post-injury status and place and acceptance within society.^34 43^ As veterans trained towards significant challenges, they utilised sport and physical activities as a form of self-medication,^43^ developing both their fitness and skills, which facilitated their re-integration into society. It also supported veterans in recognising their achievements, inspirations and direction.^34^ Engaging in outdoor adventures like hunting and fishing supported mental stimulation and development to provide meaningful life postservice.^40^

### Multiple service approach and consistency across services

Veterans who were medically discharged, bullied or who witnessed or took part in operations against enemy forces were more likely to rate multiple types of support services as necessary to improving their transition to civilian life.^3^ By contrast, veterans who served at officer ranks were more likely to state that none of the services presented would have improved their transition compared with those at lower ranks (36.5% vs 18.2%). These likely reflect differences in socioeconomic resources, health status or access to informal networks during resettlement, suggesting a need for a multiple and tailored approach to service support.

One of the largest complaints about veteran’s support services was that this varied across services and locations.^46 52^ Support during transition varied due to numerous factors, including age and service status,^3 46^ leading some to argue the need for a national commissioning framework or revenue funding for accommodation and support services.^52^ The strategies and funding framework should take account of housing need at a regional or subregional level, alongside any unmet local authority level needs. Many also expressed a need for stronger collaboration between military and non-military charities.^49^

### Monitoring systems

National level stakeholders, local authority representatives, housing specialists and dedicated service providers, have stressed the need to collect more robust data to inform service need and development.^52^ Measurement must go beyond quantitative measures, which have been referred to as rigid and encourage a fixation on individual achievements,^44^ to consider participatory research methods, ethnographies, narrative analysis and qualitative methods. These approaches will lead to community-led outputs that offer more rich and complex data to help unpick the processes occurring within support programmes.^44^ Gaps in data collection, outcome tracking and cross-system communication likely hinder continuous improvement of services.

## Discussion

### Summary

A diverse range of services and interventions designed to support Armed Forces personnel transitioning to veteran status are available across Europe and non-US Five Eyes countries. Interventions were grouped into five broad categories: art-based, sport or outdoor-based, peer- or group-based, employment or skills training and digital technology interventions. The evidence base for potential effectiveness was heterogeneous, but suggested possible benefits for mental health, wellbeing and aspects of reintegration. Sport, outdoor and arts-based approaches were each most consistently associated with improvements in wellbeing, social connection, identity and sense of purpose, with some studies reporting sustained reductions in PTSD, depression and anxiety. Digital interventions showed promise for improving mental health and reducing harmful alcohol use, while group-based psychological interventions showed more variable effects.

The success of some interventions was demonstrated through qualitative findings, which highlighted the importance of trust, psychologically safe spaces, social connection and opportunities to rebuild identity and purpose. Programmes that included structured skill-development pathways with meaningful activities, such as sport, outdoor challenges or creative practices, allowed veterans to develop transferable skills while simultaneously build confidence, social networks and a sense of purpose. For many, this reintroduced elements that were central to military life, such as routine, progression, feedback and achievement, helping to counteract the sense of loss that can accompany leaving service. Similarly, programmes that fostered camaraderie and a sense of community allowed veterans to further establish trust and mutual support with others who share similar experiences. While these findings suggest that services would benefit from including structured skill development pathways and sport or activity-based programmes that foster camaraderie, no assessment of study quality was undertaken, and it is not possible to determine whether any particular intervention category is more effective than another.

Another important observation from this review is that most evaluations focused on individual-level outcomes, including mental health, wellbeing, social connectedness and employment-related indicators. While these outcomes provide valuable evidence, relatively few examined population-level impacts or broader indicators of effectiveness. Consequently, it remains unclear whether the positive effects observed among individuals translate into measurable improvements across veteran populations, such as reductions in the prevalence of mental health problems, suicide, homelessness, unemployment, social isolation or healthcare utilisation. Future research should prioritise population-level evaluations, longitudinal study designs and linkage with administrative data where possible. Such approaches would help determine not only whether interventions benefit individual veterans, but also whether they result in meaningful and sustained improvements at a community and population level.

### Strengths and limitations

Key strengths of this review lie in its methodological rigour. We followed established methodological guidance for scoping reviews (JBI and PRISMA-ScR), included relevant grey literature to increase evidence coverage and the policy relevance of our work and used duplicate screening and extraction to enhance transparency. We also involved stakeholders in the design and interpretation of findings to bolster relevancy and ensure findings were interpreted in line with key priorities. By applying a broad inclusion strategy and integrating quantitative, qualitative and mixed-methods evidence, our review offers a holistic overview of available provision and effectiveness across physical, psychological, social and functional outcomes.

Some limitations should be noted. As a scoping review, the aim was to map and synthesise the breadth of available evidence rather than formally assess intervention effectiveness or study quality, therefore no risk-of-bias appraisal was undertaken. The evidence base was highly heterogeneous in terms of study design, populations, interventions and follow-up periods, limiting the ability to draw firm conclusions about comparative effectiveness or generalisability. In addition, many of the included studies used relatively small sample sizes which limits the extent to which causal inferences can be made regarding intervention effectiveness, or studies used qualitative designs which are better suited to identifying intervention components rather than comparative effectiveness. The restriction to studies published in the English language may have resulted in the omission of relevant evidence, and much of the evaluated evidence predates recent policy and service developments, particularly within UK primary care. It therefore remains to be seen how effectively information about services is now communicated to both healthcare providers and veterans, and what future studies will find about the impact of these more recent developments. Finally, because the review was restricted to European countries and non-US Five Eyes nations, the findings should be interpreted within these contexts.

## Conclusion

Overall, the review identifies a range of interventions and services designed to support veterans’ reintegration into civilian life. Findings suggest that approaches incorporating meaningful activity, opportunities for skill development, peer connection and support hold promise for supporting reintegration. However, the strength and consistency of the evidence varied considerably across studies, meaning findings should be interpreted as identifying potentially valuable components and approaches rather than established best practices. Future research and evaluations should prioritise robust evaluation designs, longitudinal research, consistent outcome reporting, and greater attention to implementation and equity to better inform policy and practice. Strengthening the evidence base in these areas will help determine which interventions are most effective, for whom, and under what circumstances.

## Supplementary material

10.1136/bmjopen-2026-122999 online supplemental file 1

## Data Availability

Data sharing not applicable as no datasets generated and/or analysed for this study.
