# Supplementary material for: What is the effectiveness of services and interventions for Armed Forces transitioning to veteran status, and what opportunities are there for improvement: a scoping review of health-related evidence
Source: BMJ Open. 2026 Sep 29;16(9):e122999. doi: 10.1136/bmjopen-2026-122999 (PMC13630119; doi:10.1136/bmjopen-2026-122999)
Supplement: Supplementary file 1 [file bmjopen-2026-122999.reviewer_comments.pdf]

PEER REVIEW HISTORY

BMJ Open publishes all reviews undertaken for accepted manuscripts. Reviewers are asked to complete a checklist review form and are provided with free text boxes to elaborate on their assessment. These free text comments are reproduced below.

ARTICLE DETAILS

Title (Provisional)

What is the effectiveness of services and interventions for Armed Forces transitioning to veteran status, and what opportunities are there for improvement: a scoping review of health-related evidence

Authors

Armitage, Jessica; Robinson, Sophie; Bowater, Jamie; Melendez-Torres, G.J; Barnish, Maxwell S

VERSION 1 - REVIEW

**Reviewer**	**1**
**Name**	**Fraser, John**
**Affiliation**	**Uniformed Services University of the Health Sciences F Edward Hebert School of Medicine, Physical Medicine and Rehabilitation**
**Date**	**19-Jul-2026**
**COI**	**None**

This was a scoping review evaluating the health-related services and interventions that support Armed Forces members transitioning to veteran status in European countries and non-US Five Eyes Alliance nations (United Kingdom, Canada, Australia and New Zealand). The manuscript is very well written and the study is methodologically sound. I offer the following thoughts and general comments for consideration. Thank you for the opportunity to review this highly relevant and salient study.

As a retired Naval Officer, I had a particularly tough transition as I adjusted back to civilian life due to the loss of mission, identity, and social structure following 20 years of active service. For the casual reader, they do not have the context of this experience. It would be extremely helpful to bolster the introduction and present the evidence of why these programs are needed.

While governments and non-governmental organizations have generally offered programs in contemporary times, the number and scope of these programs have changed since the OIE/OEF era began in 2001. It may be worth pointing this out in introduction and discuss the continued gaps despite the proliferation of programs. From a translation perspective, this

scoping review can serve as a needs signal for not only research to address the current knowledge gaps, but what programs are needed based on the ongoing needs of Veterans.

I appreciate the rationale for including only the UK, Commonwealth countries, and the EU in this scoping review. Given the incidence of Veteran suicide is significantly lower in these countries compared to the US, is there evidence of best practices that are translatable and that can be recommended globally?

I noticed that most of the included studies focused on individual-level health and health-related quality of life. Did you happen to come across any population-level studies of risk-factor mitigation or prevention in the Veteran community? The question I was thinking as I read your work was "are these programs making a difference?" If so, it may be worth calling out it out in the discussion. If there is a gap, expressing the need for more population-level research in this area would be appropriate.

**Reviewer**	**2**
**Name**	**Bagherian, Sajad**
**Affiliation**	**Shahrekord University**
**Date**	**20-Jul-2026**
**COI**	**None**

This manuscript addresses an important and timely topic by mapping the available evidence on interventions supporting military personnel transitioning to civilian life. The review follows established guidance for scoping reviews and includes both peer-reviewed and grey literature, providing a broad overview of existing services and interventions. The manuscript is generally well organized, and the synthesis is informative for researchers and policymakers. Nevertheless, several issues should be addressed before publication. The narrative synthesis occasionally presents intervention effectiveness more confidently than warranted by the heterogeneous evidence base. Greater distinction between evidence derived from qualitative studies and quantitative intervention studies would strengthen the interpretation. The implications for policy and practice should also be presented more cautiously, given that no formal assessment of study quality was undertaken. Finally, some methodological decisions require further clarification, and several sections could be condensed to improve readability. Overall, I believe the manuscript would be suitable for publication following minor revision.

Abstract

The abstract provides an appropriate overview of the review; however, it places considerable emphasis on the positive effects of the interventions without sufficiently acknowledging the substantial heterogeneity of the included evidence. Because no critical appraisal of study quality was performed, statements regarding effectiveness should be

phrased more cautiously. In addition, the conclusions should distinguish more clearly between mapping available evidence and demonstrating intervention effectiveness, which are different objectives in a scoping review.

Introduction

The introduction clearly establishes the importance of supporting military personnel during transition to civilian life and identifies relevant gaps in the literature. However, the rationale for limiting the review to Europe and non-US Five Eyes countries could be justified more explicitly. The authors should also explain why evidence from the United States was excluded despite the extensive veteran literature available there. Providing this justification would strengthen the conceptual framework of the review.

Materials and Methods

The methodology generally follows accepted guidance for scoping reviews and is described in sufficient detail. Nevertheless, one concern is that the absence of a formal quality appraisal limits the interpretation of intervention effectiveness, yet this issue receives relatively limited attention in the methodological discussion. The authors should also clarify how disagreements were resolved during thematic categorization of intervention types and whether any procedures were used to improve consistency in narrative synthesis. A brief explanation of how grey literature findings were integrated alongside peer-reviewed evidence would also strengthen transparency.

Results

The Results section contains a comprehensive synthesis of the available evidence, although it is considerably longer than necessary. In several places, the manuscript reports statistically significant findings from individual studies in considerable detail, which may inadvertently suggest a level of certainty that is inconsistent with the purpose of a scoping review. I encourage the authors to focus more on identifying overall patterns across studies rather than emphasizing isolated intervention outcomes. The narrative could also distinguish more consistently between quantitative evidence and qualitative observations.

Discussion

The discussion appropriately summarizes the principal findings and relates them to practical considerations for veteran support services. However, the current interpretation occasionally appears stronger than the available evidence supports. Recommendations regarding the integration of sport, arts, or skill-development programmes imply comparative effectiveness despite the absence of direct comparisons and the lack of quality assessment. The discussion would benefit from greater emphasis on the exploratory nature of the evidence and the uncertainty surrounding comparative effectiveness across intervention categories.

Limitations

The limitations section acknowledges several important issues, including study heterogeneity and the absence of risk-of-bias assessment. However, an additional limitation deserves discussion. Many included studies involved relatively small samples, uncontrolled designs, or qualitative methodologies, which substantially limits confidence in conclusions regarding intervention effectiveness. The authors should also acknowledge the possibility of publication bias and the influence of heterogeneous outcome measures on the overall synthesis.

Conclusion

The conclusion is generally consistent with the review findings but could be more cautious when referring to intervention effectiveness. Because this review maps available evidence rather than evaluates efficacy, the final statements should emphasize that current evidence identifies promising approaches requiring further rigorous evaluation rather than established best practices. This distinction is important for both researchers and policymakers.

VERSION 1 - AUTHOR RESPONSE

Reviewer: 1

Dr. John Fraser, Uniformed Services University of the Health Sciences F Edward Hebert School of Medicine, University of Kentucky

Comments to the Author:

This was a scoping review evaluating the health-related services and interventions that support Armed Forces members transitioning to veteran status in European countries and non-US Five Eyes Alliance nations (United Kingdom, Canada, Australia and New Zealand). The manuscript is very well written and the study is methodologically sound. I offer the following thoughts and general comments for consideration. Thank you for the opportunity to review this highly relevant and salient study.

Comment: Thank you for your positive comments on our review and your helpful suggestions to improve it.

As a retired Naval Officer, I had a particularly tough transition as I adjusted back to civilian life due to the loss of mission, identity, and social structure following 20 years of active service. For the casual reader, they do not have the context of this experience. It would be extremely helpful to bolster the introduction and present the evidence of why these programs are needed.

Comment: We agree that more emphasis could be added on the importance of these programs, and have therefore added the following to page 4 of the introduction, "For some, changes to identity, culture and routine can cause great difficulty during transition, and a loss of social networks can increase feelings of loneliness and present barriers to help seeking[3,4]. These problems may predict or coincide with poorer mental

health and wellbeing,"..... "Given these multifaceted challenges associated with reintegration, understanding the availability and effectiveness of interventions for veterans is essential to supporting their transition and promoting long-term health and wellbeing."

While governments and non-governmental organizations have generally offered programs in contemporary times, the number and scope of these programs have changed since the OIE/OEF era began in 2001. It may be worth pointing this out in introduction and discuss the continued gaps despite the proliferation of programs. From a translation perspective, this scoping review can serve as a needs signal for not only research to address the current knowledge gaps, but what programs are needed based on the ongoing needs of Veterans.

Comment: We would like to thank the reviewer for this helpful suggestion. We have added the following to page 4 of the introduction, "Since 2001, the landscape of veteran support has changed substantially, with both governmental and non-governmental organisations expanding the range and scope of services and interventions available to transitioning service members[8]"... "Veteran support has evolved over time in response to changing veteran needs, meaning there is a corresponding need to evaluate the effectiveness of contemporary interventions and identify areas for further service development".

I appreciate the rationale for including only the UK, Commonwealth countries, and the EU in this scoping review. Given the incidence of Veteran suicide is significantly lower in these countries compared to the US, is there evidence of best practices that are translatable and that can be recommended globally?

Comment: We agree that an important consideration is the extent to which approaches identified in the UK, Commonwealth countries and Europe may be transferable to other settings, particularly where there may be greater need. However, given distinct differences in veteran support infrastructure and healthcare systems in the US, we cannot make recommendations beyond those included in the present review. We have highlighted this in our Limitations section on page 17.

I noticed that most of the included studies focused on individual-level health and health-related quality of life. Did you happen to come across any population-level studies of risk-factor mitigation or prevention in the Veteran community? The question I was thinking as I read your work was "are these programs making a difference?" If so, it may be worth calling out it out in the discussion. If there is a gap, expressing the need for more population-level research in this area would be appropriate.

Comment: Thank you for raising this important point. Most of the studies identified evaluated outcomes at the individual level, we found comparatively little evidence examining population-level impacts. We agree that this represents an important gap in the literature and have highlighted in the Discussion by adding the following to pages 16/17, "An important observation from this review is that most evaluations focused on individual-level outcomes, including mental health, wellbeing, social connectedness, and employment-related indicators. While these outcomes provide valuable evidence, relatively few examined population-level impacts or broader indicators of effectiveness. Consequently, it remains unclear whether the positive effects observed among programme participants translate into measurable improvements across veteran populations, such as reductions in mental health problems, suicide, homelessness, unemployment, social isolation, or healthcare utilisation. Future research should prioritise population-level evaluations, longitudinal study designs, and linkage with administrative data where possible. Such approaches would help determine not only whether interventions benefit individual veterans, but also whether they result in meaningful and sustained improvements at a community and population level."

Reviewer: 2
Dr. Sajad Bagherian, Shahrekord University
Comments to the Author:
This manuscript addresses an important and timely topic by mapping the available evidence on interventions supporting military personnel transitioning to civilian life. The review follows established guidance for scoping reviews and includes both peer-reviewed and grey literature, providing a broad overview of existing services and interventions. The manuscript is generally well organized, and the synthesis is informative for researchers and policymakers. Nevertheless, several issues should be addressed before publication. The narrative synthesis occasionally presents intervention effectiveness more confidently than warranted by the heterogeneous evidence base. Greater distinction between evidence derived from qualitative studies and quantitative intervention studies would strengthen the interpretation. The implications for policy and practice should also be presented more cautiously, given that no formal assessment of study quality was undertaken. Finally, some methodological decisions require further clarification, and several sections could be condensed to improve readability. Overall, I believe the manuscript would be suitable for publication following minor revision.
Comment: We would like to thank the reviewer for their thoughtful suggestions. We appreciate the recognition of the importance of the topic and have revised the manuscript to provide a more cautious interpretation of intervention effectiveness. We have also provided additional clarification regarding methodological decisions and revised several sections to improve readability and conciseness. We believe these changes have strengthened the manuscript.

Abstract

The abstract provides an appropriate overview of the review; however, it places considerable emphasis on the positive effects of the interventions without sufficiently acknowledging the substantial heterogeneity of the included evidence. Because no critical appraisal of study quality was performed, statements regarding effectiveness should be phrased more cautiously. In addition, the conclusions should distinguish more clearly between mapping available evidence and demonstrating intervention effectiveness, which are different objectives in a scoping review.

Comment: We have softened the language of our conclusion statement and added that "the strength and consistency of the evidence varied considerably across studies, meaning findings should be interpreted as identifying potentially valuable components and approaches rather than established best practices".

Introduction

The introduction clearly establishes the importance of supporting military personnel during transition to civilian life and identifies relevant gaps in the literature. However, the rationale for limiting the review to Europe and non-US Five Eyes countries could be justified more explicitly. The authors should also explain why evidence from the United States was excluded despite the extensive veteran literature available there. Providing this justification would strengthen the conceptual framework of the review.

Comment: Thank you for this point. The US was excluded because it has a large and distinct veteran support infrastructure, healthcare system, and body of veteran-specific research that may not be transferable to other national contexts. Existing reviews have predominantly synthesised evidence from the US, meaning less is known about interventions available in European and non-US Five Eyes nations. The revised text on page 4 more explicitly highlights this, "The US has a distinct veteran support landscape which involves the Department of Veterans Affairs, and differing eligibility pathways for benefits and healthcare. This is likely to impact military culture and experiences both during and post-service[17], meaning findings from US studies may not be directly transferable to other contexts. Given that existing reviews are heavily dominated by US evidence, an important gap remains in synthesising evidence from settings with comparable systems of veteran support and transition."

Materials and Methods

The methodology generally follows accepted guidance for scoping reviews and is described in sufficient detail. Nevertheless, one concern is that the absence of a formal quality appraisal limits the interpretation of intervention effectiveness, yet this issue receives relatively limited attention in the methodological discussion. The authors should also clarify how disagreements were resolved during thematic categorization of

intervention types and whether any procedures were used to improve consistency in narrative synthesis. A brief explanation of how grey literature findings were integrated alongside peer-reviewed evidence would also strengthen transparency.

Comment: Thank you for these helpful suggestions. We have added that 'a formal quality appraisal was not conducted' to page 6 and have added to the Methods section on page 7 to clarify that "Intervention types were identified and categorised inductively during data synthesis based on their primary aims and characteristics. Any uncertainties or disagreements regarding categorisation were resolved through discussion and consensus". We have also added further detail on the procedures used to enhance consistency during the narrative synthesis, including the use of a "predefined data extraction framework". We have now also clarified how findings from grey literature sources were incorporated alongside peer-reviewed studies on page 7.

Results

The Results section contains a comprehensive synthesis of the available evidence, although it is considerably longer than necessary. In several places, the manuscript reports statistically significant findings from individual studies in considerable detail, which may inadvertently suggest a level of certainty that is inconsistent with the purpose of a scoping review. I encourage the authors to focus more on identifying overall patterns across studies rather than emphasizing isolated intervention outcomes. The narrative could also distinguish more consistently between quantitative evidence and qualitative observations.

Comment: We have the Results section to reduce some emphasis on individual study findings and improve the distinction between quantitative and qualitative findings. We have retained reporting of findings from some individual studies where these contribute to understanding the scope of the results and their effect sizes. Our intention is not to imply certainty regarding effectiveness, but rather to provide readers with sufficient detail to understand how outcomes have been reported across different interventions and outcomes.

Discussion

The discussion appropriately summarizes the principal findings and relates them to practical considerations for veteran support services. However, the current interpretation occasionally appears stronger than the available evidence supports. Recommendations regarding the integration of sport, arts, or skill-development programmes imply comparative effectiveness despite the absence of direct comparisons and the lack of quality assessment. The discussion would benefit from greater emphasis on the exploratory nature of the evidence and the uncertainty surrounding comparative effectiveness across intervention categories.

Comment: Thank you for this point. We have revised the Discussion on page 16 to acknowledge the absence of direct comparisons between intervention types and highlight that no formal quality assessment was undertaken. We have edited the language to avoid statements that could be interpreted as implying comparative effectiveness.

Limitations

The limitations section acknowledges several important issues, including study heterogeneity and the absence of risk-of-bias assessment. However, an additional limitation deserves discussion. Many included studies involved relatively small samples, uncontrolled designs, or qualitative methodologies, which substantially limits confidence in conclusions regarding intervention effectiveness. The authors should also acknowledge the possibility of publication bias and the influence of heterogeneous outcome measures on the overall synthesis.

Comment: We have added to the Limitations section about the study sample sizes and qualitative methods, noting that "many of the included studies used relatively small sample sizes which limits the extent to which causal inferences can be made regarding intervention effectiveness, or studies used qualitative designs which are better suited to identifying intervention components rather than comparative effectiveness". The heterogeneous nature of the evidence base is already included in the limitations section and whilethe possibility of publication bias cannot be excluded, several included studies reported mixed or non-significant findings, suggesting that the available evidence was not exclusively composed of positive outcomes.

Conclusion

The conclusion is generally consistent with the review findings but could be more cautious when referring to intervention effectiveness. Because this review maps available evidence rather than evaluates efficacy, the final statements should emphasize that current evidence identifies promising approaches requiring further rigorous evaluation rather than established best practices. This distinction is important for both researchers and policymakers.

Comment: Thank you for this comment. We have edited the Conclusion on page 18 to adopt more cautious language, and have added the following to emphasis the promising approaches that require further evaluation, "the review identifies a range of interventions and services designed to support veterans' reintegration into civilian life. Findings suggest that approaches incorporating meaningful activity, opportunities for skill development, peer connection and support hold promise for supporting reintegration. However, the strength and consistency of the evidence varied considerably across studies, meaning findings should be interpreted as identifying

potentially valuable components and approaches rather than established best practices”. We also note that “Strengthening the evidence base in these areas will help determine which interventions are most effective, for whom, and under what circumstances.”.

VERSION 2 - REVIEW

**Reviewer**	**2**
**Name**	**Bagherian, Sajad**
**Affiliation**	**Shahrekord University**
**Date**	**03-Sep-2026**
**COI**	

The revised manuscript addresses the major concerns raised in the previous review and is substantially improved. The research question, methodology, eligibility criteria, and synthesis approach are now sufficiently described, and the conclusions are appropriately more cautious given the heterogeneity of the evidence. The limitations of the scoping review are also adequately acknowledged.

I have only minor concerns that should be addressed before publication. In particular, the authors should reconcile the numerical inconsistencies between the Results section and the PRISMA flow diagram and undertake a final careful proofreading of the manuscript to correct remaining grammatical, typographical, and editorial errors. In addition, some statements regarding intervention effectiveness could be phrased more cautiously to remain consistent with the heterogeneous evidence base and the absence of formal risk-of-bias assessment.

VERSION 2 - AUTHOR RESPONSE

Reviewer: 2
Dr. Sajad Bagherian, Shahrekord University
Comments to the Author:
The revised manuscript addresses the major concerns raised in the previous review and is substantially improved. The research question, methodology, eligibility criteria, and synthesis approach are now sufficiently described, and the conclusions are appropriately more cautious given the heterogeneity of the evidence. The limitations of the scoping review are also adequately acknowledged.

I have only minor concerns that should be addressed before publication. In particular, the authors should reconcile the numerical inconsistencies between the Results

section and the PRISMA flow diagram and undertake a final careful proofreading of the manuscript to correct remaining grammatical, typographical, and editorial errors. In addition, some statements regarding intervention effectiveness could be phrased more cautiously to remain consistent with the heterogeneous evidence base and the absence of formal risk-of-bias assessment.

Response:

We would like to thank the reviewer for their positive comments on our revised manuscript. We have now reconciled the numerical inconsistencies between the Results section and the PRISMA flow diagram and have conducted a final proofread to correct any remaining errors. We have also softened statements around effectiveness.
