# Supplementary material for: What is the effectiveness of services and interventions for Armed Forces transitioning to veteran status, and what opportunities are there for improvement: a scoping review of health-related evidence
Source: BMJ Open. 2026 Sep 29;16(9):e122999. doi: 10.1136/bmjopen-2026-122999 (PMC13630119; doi:10.1136/bmjopen-2026-122999)
Supplement: Supplementary file 2 [file bmjopen-16-9-s001.docx]

**Supplementary file**

**Supplementary File 1. PRISMA-ScR checklist for scoping reviews**

| **SECTION** | **ITEM** | **PRISMA-ScR CHECKLIST ITEM** | **REPORTED ON PAGE #** |
| --- | --- | --- | --- |
| **TITLE** | | | |
| Title | 1 | Identify the report as a scoping review. | 1 |
| **ABSTRACT** | | | |
| Structured summary | 2 | Provide a structured summary that includes (as applicable): background, objectives, eligibility criteria, sources of evidence, charting methods, results, and conclusions that relate to the review questions and objectives. | 2-3 |
| **INTRODUCTION** | | | |
| Rationale | 3 | Describe the rationale for the review in the context of what is already known. Explain why the review questions/objectives lend themselves to a scoping review approach. | 4-5 |
| Objectives | 4 | Provide an explicit statement of the questions and objectives being addressed with reference to their key elements (e.g., population or participants, concepts, and context) or other relevant key elements used to conceptualize the review questions and/or objectives. | 5 |
| **METHODS** | | | |
| Protocol and registration | 5 | Indicate whether a review protocol exists; state if and where it can be accessed (e.g., a Web address); and if available, provide registration information, including the registration number. | 6 |
| Eligibility criteria | 6 | Specify characteristics of the sources of evidence used as eligibility criteria (e.g., years considered, language, and publication status), and provide a rationale. | 6-7 |
| Information sources* | 7 | Describe all information sources in the search (e.g., databases with dates of coverage and contact with authors to identify additional sources), as well as the date the most recent search was executed. | 6 |
| Search | 8 | Present the full electronic search strategy for at least 1 database, including any limits used, such that it could be repeated. | 6, Supplemental File 2 |
| Selection of sources of evidence† | 9 | State the process for selecting sources of evidence (i.e., screening and eligibility) included in the scoping review. | 6-7 |
| Data charting process‡ | 10 | Describe the methods of charting data from the included sources of evidence (e.g., calibrated forms or forms that have been tested by the team before their use, and whether data charting was done independently or in duplicate) and any processes for obtaining and confirming data from investigators. | 6-7 |
| Data items | 11 | List and define all variables for which data were sought and any assumptions and simplifications made. | 6-7 |
| Critical appraisal of individual sources of evidence§ | 12 | If done, provide a rationale for conducting a critical appraisal of included sources of evidence; describe the methods used and how this information was used in any data synthesis (if appropriate). | N/A |
| Synthesis of results | 13 | Describe the methods of handling and summarizing the data that were charted. | 7 |
| **RESULTS** | | | |
| Selection of sources of evidence | 14 | Give numbers of sources of evidence screened, assessed for eligibility, and included in the review, with reasons for exclusions at each stage, ideally using a flow diagram. | 8 |
| Characteristics of sources of evidence | 15 | For each source of evidence, present characteristics for which data were charted and provide the citations. | 8, Supplemental Files 3-4, 8 |
| Critical appraisal within sources of evidence | 16 | If done, present data on critical appraisal of included sources of evidence (see item 12). | N/A |
| Results of individual sources of evidence | 17 | For each included source of evidence, present the relevant data that were charted that relate to the review questions and objectives. | 8-14, Supplemental Files 5-6 |
| Synthesis of results | 18 | Summarize and/or present the charting results as they relate to the review questions and objectives. | Figure 1 |
| **DISCUSSION** | | | |
| Summary of evidence | 19 | Summarize the main results (including an overview of concepts, themes, and types of evidence available), link to the review questions and objectives, and consider the relevance to key groups. | 15 |
| Limitations | 20 | Discuss the limitations of the scoping review process. | 16 |
| Conclusions | 21 | Provide a general interpretation of the results with respect to the review questions and objectives, as well as potential implications and/or next steps. | 16-17 |
| **FUNDING** | | | |
| Funding | 22 | Describe sources of funding for the included sources of evidence, as well as sources of funding for the scoping review. Describe the role of the funders of the scoping review. | 18 |

**Supplementary File 2. Full search strategies**

| **Database** | **Search date** | **Hits** |
| --- | --- | --- |
| Medline (Ovid) | 6/11/25 | 447 |
| Embase (Ovid) | 6/11/25 | 564 |
| APA PsycInfo (Ovid) | 6/11/25 | 2450 |
| Cochrane (Wiley) | 6/11/25 | 826 |
| Web of Science (Clarivate) | 7/11/25 | 626 |
| ASSIA (ProQuest) | 7/11/25 | 1511 |
| PTSD Pubs (Proquest) | 7/11/25 | 1879 |
| Total records |  | 9123 |
| Duplicate records |  | 2109 |
| **Final total to screen** |  | **7014** |

**Ovid MEDLINE(R) ALL <1946 to November 05, 2025>**

1	Veterans/	25000

2	veteran*.ti,ab,kf.	51834

3	((army or military or marine* or soldier* or navy or force* or "service person*" or "service people" or "service man" or "service men" or "service woman" or "service women" or troop* or "merchant mariner" or gurkha*) adj3 (ex or long or former or previous* or length or past)).ti,ab,kf.	8228

4	or/1-3	62340

5	(discharge* or leav* or quit* or transition* or dismiss* or release* or fire* or sack* or 'let go' or remove or 'lay off' or retire*).ti,ab,kf.	2503065

6	Personnel Turnover/	6681

7	Retirement/	11875

8	5 or 6 or 7	2511155

9	exp australasia/ or exp europe/ or exp united kingdom/ or exp Russia/ or exp Canada/	2047213

10	("five eyes" or "5 eyes").ti,ab,kf.	5423

11	European Union/	18724

12	("national health service" or nhs).ti,ab,kf.	57229

13	(europe* or "united kingdom" or uk or "great britain" or england or scotland or wales or ireland or canada or "new zealand" or australia or australasia or france or germany or spain or portugal or sweden or norway or finland or denmark or belgium or austria or germany or czech* or greece or iceland or italy or baltic or estonia or hungary or latvia or lithuania or luxembourg or croatia or netherlands or poland or scandinavia or slovakia or slovenia or switzerland or turkey or russia or ukraine or romania or belarus or bulgaria or serbia or bosnia or moldova or lithuania or albania or latvia or macedonia or estonia or montenegro or malta or andorra or liechtenstein or monaco or "san marino" or "holy see" or vatican or cyprus or gibraltar or caucasus or kazakhstan).ti,ab,kf.	1699605

14	9 or 10 or 11 or 12 or 13	2913795

15	4 and 8 and 14	447

**Embase <1974 to 2025 November 04>**

1	Veteran/	43968

2	veteran*.ti,ab,kf.	72978

3	((army or military or marine* or soldier* or navy or force* or "service person*" or "service people" or "service man" or "service men" or "service woman" or "service women" or troop* or "merchant mariner" or gurkha*) adj3 (ex or long or former or previous* or length or past)).ti,ab,kf.	9146

4	or/1-3	84257

5	(discharge* or leav* or quit* or transition* or dismiss* or release* or fire* or sack* or 'let go' or remove or 'lay off' or retire*).ti,ab,kf.	3203790

6	exp labor market exit/	26293

7	5 or 6	3213834

8	exp "Australia and New Zealand"/ or exp europe/ or exp united kingdom/ or exp Russia/ or exp Canada/	2418938

9	("five eyes" or "5 eyes").ti,ab,kf.	7524

10	European Union/	34307

11	("national health service" or nhs).ti,ab,kf.	92420

12	(europe* or "united kingdom" or uk or "great britain" or england or scotland or wales or ireland or canada or "new zealand" or australia or australasia or france or germany or spain or portugal or sweden or norway or finland or denmark or belgium or austria or germany or czech* or greece or iceland or italy or baltic or estonia or hungary or latvia or lithuania or luxembourg or croatia or netherlands or poland or scandinavia or slovakia or slovenia or switzerland or turkey or russia or ukraine or romania or belarus or bulgaria or serbia or bosnia or moldova or lithuania or albania or latvia or macedonia or estonia or montenegro or malta or andorra or liechtenstein or monaco or "san marino" or "holy see" or vatican or cyprus or gibraltar or caucasus or kazakhstan).ti,ab,kf.	2643751

13	8 or 9 or 10 or 11 or 12	3728607

14	4 and 7 and 13	564

**APA PsycInfo <1806 to October 2025 Week 5>**

1	Military Veterans/	18910

2	veteran*.tw.	30038

3	((army or military or marine* or soldier* or navy or force* or "service person*" or "service people" or "service man" or "service men" or "service woman" or "service women" or troop* or "merchant mariner" or gurkha*) adj3 (ex or long or former or previous* or length or past)).tw.	1792

4	or/1-3	18910

5	(discharge* or leav* or quit* or transition* or dismiss* or release* or fire* or sack* or 'let go' or remove or 'lay off' or retire*).tw.	348314

6	Employee Turnover/	7962

7	Retirement/	6034

8	5 or 6 or 7	353687

9	4 and 8	2450

**Cochrane Library**

Date Run:	06/11/2025

ID	Search	Hits

#1	veteran*:ti,ab	7874

#2	(army or military or marine* or soldier* or navy or force* or "service person" or "service man" or "service woman" or troop* or "merchant mariner") near/3 (ex or long or former or previous* or length or past):ti,ab	271

#3	MeSH descriptor: [Veterans] explode all trees	1817

#4	#1 or #2 or #3	8239

#5	(discharge* or leav* or quit* or transition* or dismiss* or release* or fire* or sack* or 'let go' or remove or 'lay off' or retire*):ti,ab	144490

#6	MeSH descriptor: [Personnel Turnover] explode all trees	61

#7	MeSH descriptor: [Retirement] explode all trees	97

#8	#5 or #6 or #7	144538

#9	#4 and #8	826

**Web of Science Core Collection** 	**7/11/25**

9. #4 AND #7 AND #8 	626

8. TS=(discharge* or leav* or quit* or transition* or dismiss* or release* or fire* or sack* or 'let go' or remove or 'lay off' or retire*) 	6,901,651

7. #5 OR #6	4,969,589

6. TS=("national health service" or nhs or "five eyes" or "5 eyes") 	60,181

5. TS=(europe* or "united kingdom" or uk or "great britain" or england or scotland or wales or ireland or canada or "new zealand" or australia or australasia or france or germany or spain or portugal or sweden or norway or finland or denmark or belgium or austria or germany or czech* or greece or iceland or italy or baltic or estonia or hungary or latvia or lithuania or luxembourg or croatia or netherlands or poland or scandinavia or slovakia or slovenia or switzerland or turkey or russia or ukraine or romania or belarus or bulgaria or serbia or bosnia or moldova or lithuania or albania or latvia or macedonia or estonia or montenegro or malta or andorra or liechtenstein or monaco or "san marino" or "holy see" or vatican or cyprus or gibraltar or caucasus or kazakhstan)	4,933,951

4. #1 or #2 or #3 	75,461

3. TS=(veteran*)	74,614

2.TS=("ex army" or "ex military" or "ex marine*" or "ex soldier*" or "ex navy" or "ex force*" or "ex service person*" or "ex service people" or "ex service man" or "ex service men" or "ex service woman" or "ex service women" or "ex troop*" or "ex merchant mariner" or "ex gurkha")	326

1. TS=("former army" or "former military" or "former marine*" or "former soldier*" or "former navy" or "former force*" or "former service person*" or "former service people" or "former service man" or "former service men" or "former service woman" or "former service women" or "former troop*" or "former merchant mariner" or "former gurkha")  723

**Applied Social Sciences Index & Abstracts (ASSIA)**	**7/11/25**

((ti,ab(discharge* OR leav* OR quit* OR transition* OR dismiss* OR release* OR fire* OR sack* OR 'let go' OR remove OR 'lay off' OR retire*) OR MAINSUBJECT.EXACT.EXPLODE("Retirement") OR MAINSUBJECT.EXACT.EXPLODE("Employee turnover")) AND (ti,ab("ex army" OR "ex military" OR "ex marine*" OR "ex soldier*" OR "ex navy OR " ex force* " OR " ex service person " OR " ex service personal " OR " ex service personnel " OR " ex service persons " OR " ex service man " OR " ex service woman " OR " ex troop* " OR " ex merchant mariner " OR " ex Gurkha ") OR (" former army " OR " former military " OR " former marine* " OR " former soldier* " OR " former navy OR "ex force*" OR "former service person" OR "former service personal" OR "former service personnel" OR "former service persons" OR "former service man" OR "former service woman" OR "former troop*" OR "former merchant mariner" OR "former Gurkha")) OR ti.ab (veteran*) OR MAINSUBJECT.EXACT("Veterans")) AND (ti,ab(national health service* OR nhs* OR "five eyes" OR "5 eyes") OR (europe* OR "united kingdom" OR uk OR "great britain" OR england OR scotland OR wales OR ireland OR canada OR "new zealand" OR australia OR australasia OR france OR germany OR spain OR portugal OR sweden OR norway OR finland OR denmark OR belgium OR austria OR germany OR czech* OR greece OR iceland OR italy OR baltic OR estonia OR hungary OR latvia OR lithuania OR luxembourg OR croatia OR netherlands OR poland OR scandinavia OR slovakia OR slovenia OR switzerland OR turkey OR russia OR ukraine OR romania OR belarus OR bulgaria OR serbia OR bosnia OR moldova OR lithuania OR albania OR latvia OR macedonia OR estonia OR montenegro OR malta OR andorra OR liechtenstein OR monaco OR "san marino" OR "holy see" OR vatican OR cyprus OR gibraltar OR caucasus OR kazakhstan))	1511 results

**PTSD Pubs**	**7/11/25**

((ti,ab(discharge* OR leav* OR quit* OR transition* OR dismiss* OR release* OR fire* OR sack* OR 'let go' OR remove OR 'lay off' OR retire*) OR MAINSUBJECT.EXACT.EXPLODE("Retirement")) AND (ti,ab("ex army" OR "ex military" OR "ex marine*" OR "ex soldier*" OR "ex navy OR " ex force* " OR " ex service person " OR " ex service personal " OR " ex service personnel " OR " ex service persons " OR " ex service man " OR " ex service woman " OR " ex troop* " OR " ex merchant mariner " OR " ex Gurkha ") OR (" former army " OR " former military " OR " former marine* " OR " former soldier* " OR " former navy OR "ex force*" OR "former service person" OR "former service personal" OR "former service personnel" OR "former service persons" OR "former service man" OR "former service woman" OR "former troop*" OR "former merchant mariner" OR "former Gurkha")) OR ti.ab (veteran*) OR MAINSUBJECT.EXACT.EXPLODE("Veterans")) AND (ti,ab(national health service* OR nhs* OR "five eyes" OR "5 eyes") OR (europe* OR "united kingdom" OR uk OR "great britain" OR england OR scotland OR wales OR ireland OR canada OR "new zealand" OR australia OR australasia OR france OR germany OR spain OR portugal OR sweden OR norway OR finland OR denmark OR belgium OR austria OR germany OR czech* OR greece OR iceland OR italy OR baltic OR estonia OR hungary OR latvia OR lithuania OR luxembourg OR croatia OR netherlands OR poland OR scandinavia OR slovakia OR slovenia OR switzerland OR turkey OR russia OR ukraine OR romania OR belarus OR bulgaria OR serbia OR bosnia OR moldova OR lithuania OR albania OR latvia OR macedonia OR estonia OR montenegro OR malta OR andorra OR liechtenstein OR monaco OR "san marino" OR "holy see" OR vatican OR cyprus OR gibraltar OR caucasus OR kazakhstan)) 	1879 results

**Web searches**

| **Organisation** | **Number of hits** |
| --- | --- |
| UK Ministry of Defence – Veterans UK  www.gov.uk/government/organisations/veterans-uk | 0 |
| Combat Stress  [http://www.combatstress.org](http://www.combatstress.org/) | 4 reports |
| Kings Centre for Military Health Research  https://kcmhr.org | 4 reports |
| Westminster Centre for Research in Veterans  [www.chester.ac.uk/research/research-and-knowledge-exchange-institutes-rkeis/research-centres/westminster-centre-for-research-in-veterans](http://www.chester.ac.uk/research/research-and-knowledge-exchange-institutes-rkeis/research-centres/westminster-centre-for-research-in-veterans) | 4 reports |
| Centre for Evidence for the Armed Forces Community  [https://www.centreforevidence.org](https://www.centreforevidence.org/) | 55 reports |
| Centre for Military Women’s Research  <https://www.centreformilitarywomensresearch.com/> | 12 reports |
| Veterans and Families Institute for Military Social Research  <https://www.aru.ac.uk/veterans-and-families-institute> | 1 reports |

##### **Supplementary File 3. Study design of included studies**

| Study | Data years | Design | Population | Intervention | Comparator | Outcomes | Sample size | Analysis method |
| --- | --- | --- | --- | --- | --- | --- | --- | --- |
| Ackley 2023 | Not stated | Semi-structured interviews | Military veterans or family members | Theatre programme | None | Psychosocial outcomes | 6 | Thematic analysis |
| Albertson 2019 | 1976 to 2011 | Qualitative study | Military veterans | Peer support group | None | Sense of belonging | 35 | Thematic analysis |
| Alichniewicz 2025 | 2021 to 2023 | Preliminary usability study | Military veterans | Self-directed internet-based programme | None | Uptake and user experience | 959 (273 for the intervention) | Statistical and thematic analysis |
| Bailey 2023 | Up to 2019 | Qualitative interviews | Service providers to military veterans | Service charities | None | Access to services | 38 | Thematic analysis (iterative approach) |
| Belliveau 2019 | 2015 to 2016 | Survey (qualitative) | Military veterans | Theatre programme | None | Mental health issues | 6 | Thematic analysis |
| Belliveau 2017 | 2015 to 2016 | Narrative account | Military veterans | Theatre programme | None | Experiences of the intervention | Not stated | Narrative analysis (qualitative) |
| Brittain 2012 | 2011 | Life course analysis based on media coverage | Military veterans | None | None | Experiences of using sport as part of rehabilitation | Not stated | Qualitative analysis |
| Carins 2025 | 2023 to 2024 | Qualitative study (based on workshops) | Military veterans and healthcare professionals | Co-design workshop | None | Ideas for new programmes to reduce alcohol abuse | 19 (12 veterans and 7 healthcare professionals) | Content analysis (qualitative) |
| Cox 2019 | Not stated | Single-armed trial | Military veterans | Group transition programme | None | Impact on PTSD, perceived social support | 218 | Path analysis |
| Edwards 2022 | Not stated | Interviews (qualitative and quantitative data) | Military veterans | Veteran support service | None | Experience and needs post-discharge | 175 | Qualitative inductive analysis and descriptive statistics |
| Forces in Mind Trust 2021 | 2019 | Mixed methods | Military veterans | Veterans’ advocacy service | None | Impact of service on veterans and their families | 199 | Social return on investment analysis |
| Forces in Mind Trust 2013 | Not stated | Interviews, workshop, survey and focus groups | Military veterans and relevant stakeholders | None | None | Transition issues | 29 (veterans interviewed) | Descriptive |
| Gati 2013 | Not stated | Pretest-posttest design using questionnaires | Military service members | 5-day workshop aimed to help transition to civilian life | None | Career development and decision-making | 1315 | Statistical analysis |
| Gillin 2021 | 2015 to 2018 | Mixed methods | Military veterans | Intervention 1: Spouse employment support. Intervention 2: MoD programme to help early leavers | None | Intervention 1: Spouses’ employment prospects. Intervention 2: Ease of civilian transition for early leavers | Intervention 1: 111 survey responses and 42 interviews Intervention 2: 5,399 survey responses. | Survey analysis (descriptive statistics and qualitative) |
| Godier-McBard 2021 | 2020 | Interviews and stakeholder workshops | Military veterans and stakeholders | None | None | To learn about the needs of female veterans and to develop priorities | 9 veterans, 13 stakeholders | Thematic analysis |
| Harper 2014 | Not stated | Longitudinal mixed methods | Military veterans | Outdoor and experiential learning program | None | Self-confidence, goal setting, resilience, problem solving | 54 (quantitative analysis), 12 (qualitative analysis) | Statistical and phenomenological analysis |
| Healthwatch Hertfordshire 2021 | 2020 | Online survey, focus group and interviews | Military veterans and local NHS organisations | None | None | Healthcare needs and experiences of veterans in Hertfordshire | 87 (veterans surveyed), 7 (veterans interviewed), 8 stakeholders (interviewed) | Descriptive statistics (survey), template analysis (interviews) |
| Heaver 2018 | 2017 | Online survey and case study interviews | Military veterans | None | None | Experiences of civilian transition and the needs of families during transition | 168 (survey), 21 (interviews) | Not stated |
| Hooks 2023 | Not stated | Interviews, surveys and stakeholder workshops | Military veterans | None | None | Experiences of female veterans in the civilian transition process | 85 | Thematic analysis |
| Hutcheon 2024 | 2022 | Semi-structured interviews and survey | Military veterans | Employment training programme | None | Experiences of veterans pre- and post- programme | 10 | Thematic analysis |
| Jones/ Quilgars 2014/2018 | 2013 to 2017 | Series of interviews with veterans, review of services, case studies of providers, roundtable (mixed methods) | Military veterans, local authorities, service providers | Services for single veterans | None | Veteran experiences related to housing pathways and available services | 35 veterans (15 interviewed) | Qualitative (no details provided). Quantitative (descriptive) |
| Mackintosh 2025 | Not stated | Interviews | Physically disabled military veterans | Participating in sports activities | None | Role of sport and physical activity | 8 | Narrative analysis |
| Meierdirk 2024 | Not stated | Online semi-structured interviews | Military veterans | Education relevant to working in the construction industry | None | Transition to employment | 9 | Constant comparison analysis |
| Metcalf 2022 | 2018 to 2020 | A two arm-parallel group RCT | Military service members and veterans | Attention-control training | Placebo attention training | Level of PTSD, functional impairment | 69 | Statistical analysis via analysis of covariance (ANCOVA) |
| Moran 2024 | 2021 to 2023 | Nonrandomised controlled longitudinal study | Military service members and veterans (84.5%) | Surf therapy | No intervention | PTSD, depression, anxiety | 116 | Descriptive statistics and statistical modelling |
| O'Connor 2017 | Not stated | Case study (qualitative) | Military veterans and family members | Theatre programme | None | Feedback on the programme and experiences of civilian transition | 8 veterans and 5 family members | Qualitative |
| O’Connor 2015 | 2012 | Practice-based report (qualitative) | Military veterans | Theatre programme | None | Process and impact of the programme | 13 (of which 8 performed) | Qualitative |
| O’Hanlon 2024 | 2019-2020 | Ethnographic approach underpinned by social constructivism (qualitative) | Staff and stakeholders | Sport programme | None | Realities and challenges of administering the programme | 14 staff and stakeholders | Dialogical narrative analysis |
| Parkes 2023/2022 | 2021 | Feasibility RCT | Military veterans | Mental health smartphone app | An app containing only signposting information | Recruitment and retention; trial procedures and outcome measures; and acceptability and usability | 791 | Descriptive statistics; t-tests and chi-square tests |
| Romanuik 2024 | 2021 | Within-participants, non-controlled, longitudinal design (mixed methods) | Military veterans | Group programme using cognitive-behavioural and acceptance and commitment therapy techniques | None | Military-civilian adjustment and reintegration measure (M-CARM) | 24 | ANOVA; qualitative analysis (appears to be thematic analysis) |
| Waldhauser 2021 | 2019 | Semi-structured interviews | Military veterans | “Ball hockey” programme | None | Aspects of the programme that the participants experienced and considered important | 12 | Reflexive thematic analysis |
| Westwood 2010 | Not stated | Quantitative and qualitative methods in a repeated measures quasi-experimental design | Military service members | Veterans transition programme | None | PTSD, depression, self-worth, experience of re-adjustment to home life, what was helpful and a hindrance in programme | 18 | Descriptive statistics, Critical Incident Technique |
| Williamson 2022 | 2020-2021 | Mixed methods pilot then RCT | Military veterans and experts | Smartphone alcohol app | RCT: access only to questionnaire and feedback modules of the app | Pilot: usability. RCT: alcohol consumption, AUDIT score - tool to measure risky drinking, engagement, usability, adverse events | Pilot: 8 veterans and 8 experts; RCT: 123 veterans | Descriptive statistics; thematic analysis (pilot only); statistical analysis – probably regression but not stated (RCT only) |
| Wood 2023 | 2022-2023 | Semi-structured interviews and focus groups | Military veterans and mental healthcare professionals | Guidance for mental healthcare professionals (MHCPs) and recommendations for commissioners | Current practice | Mental healthcare support needs and experiences | 48 veterans and 12 mental health professionals | Reflexive thematic analysis |

Abbreviations: MoD, Ministry of Defence; PTSD, Posttraumatic Stress Disorder; RCT, Randomised Controlled Trial

##### **Supplementary File 4. Participant profiles of included studies**

| Study | Country | Service branch | % Female | Before or after discharge | Time after discharge | Type of discharge | Mental health issues | Other key details |
| --- | --- | --- | --- | --- | --- | --- | --- | --- |
| Ackley 2023 | UK | Army, RAF, FFL | 16.7% | After | Not stated | Not stated | Emotional problems (or physical health issues) | Had participated in a theatre programme |
| Albertson 2019 | UK | Army, Navy, RAF | Not stated (some females) | After | Not stated | Not stated | Not stated | None |
| Alichniewicz 2025 | Australia | ADF | 26% | After | Not stated | Not stated | Not stated | None |
| Bailey 2023 | UK | Not stated | Female access to services | After | Not stated | Not stated | Not stated | Interviews with service providers |
| Belliveau 2019 | International (two-thirds Canada) | Not stated | All male | After | Not stated | Not stated | Not stated | Involved in a theatrical programme |
| Belliveau 2017 | Canada | Not stated | All male | After | Not stated | Not stated | Not stated | Returning veterans |
| Brittain 2012 | UK, Australia, Canada and USA | Not stated | Not stated | After | Not stated | Not stated | Not stated | Experienced a sudden traumatic disability |
| Carins 2025 | New Zealand | Not stated | 17% | After | Not stated | Not stated | Not stated | None |
| Cox 2019 | Canada | Army, Navy and air force | 11% | After | Not stated | Not stated | 97.7% had probable PTSD | None |
| Edwards 2022 | UK | Army, Navy and RAF | 100% | After | Not stated | Not stated | Not stated | None |
| Forces in Mind Trust 2021 | UK (living in England) | Not stated | Not stated | After | Not stated | Not stated | Not stated | One-to-one support via Veterans’ Advocacy People |
| Forces in Mind Trust 2013 | UK | Army, Navy and RAF | Not stated | After | Not stated | Various types of discharge | Not stated | None |
| Gati 2013 | Israel | Not stated | 50.9% | Before | NA | NA | Not stated | Young adults, approaching end of mandatory military service |
| Gillin 2021 | UK | Army, Navy and air force | Not stated | After | Not stated | Not stated | Not stated | Study 1: limbless |
| Godier-McBard 2021 | UK | Army, Navy and air force | 77% | After | Not stated | Not stated | Not stated | None |
| Harper 2014 | Canada | Not stated | 13% | After | Not stated | Not stated | 22.2% had mental health issues | Enrolled on Outward Bound Canada veterans course |
| Healthwatch Hertfordshire 2021 | UK | Army, Navy and air force | 13% | After | 75% had left the AF over 16 years ago | Not stated | Not stated | None |
| Heaver 2018 | UK | Army, Navy and air force | Not stated | After | Not stated | Not stated | Not stated | None |
| Hooks 2023 | UK | Army, Navy and air force | 100% | After | Not stated | Not stated | Not stated | None |
| Hutcheon 2024 | UK | Army and Navy | All male | After | Not stated | Not stated | Not stated | Existing employees in one particular company |
| Jones/ Quilgars 2014/2018 | UK | Army, Navy and air force | Not stated | After | Not stated | Not stated | Not stated | Single (living on their own) |
| Mackintosh 2025 | UK | Army, Navy and air force | 62.5% | After | Not stated | Not stated | Not stated | Physically disabled |
| Meierdirk 2024 | UK | Army and Navy | Not stated | After | Not stated | Not stated | Not stated | Attended a particular construction course |
| Metcalf 2022 | Australia | Not stated | 18.6% | Before and after | From 4 months pre-discharge to 4 months post-discharge | Not stated | Not stated | Had served in the military for at least 12 months |
| Moran 2024 | Australia | Not stated | 12% | Before and after | Not stated | Not stated | Not stated | None |
| O'Connor 2017 | UK | Not stated | Not stated | After | Not stated | Not stated | Not stated | None |
| O’Connor 2015 | UK | Not stated | All male (veterans appearing on stage) | After | Not stated | Not stated | Not stated | None |
| O’Hanlon 2024 | UK | Not stated | NA | After | NA | NA | Not stated | Participating in a veterans sport programme |
| Parkes 2023/2022 | UK | Army (64%), Navy and air force | All male | After | At least 2 years | Not stated | Mental health distress (scored 2 or more on the GHQ-12) – not currently receiving formal mental health treatment | Served at least 2 years in the AF |
| Romanuik 2024 | Australia | Army (79.2%), Navy and air force | 8.3% | After | Less than 1 year to 42 years | Various (33% medical discharge) | Potential difficulties in at least three of the five M-CARM factors | Self-reported difficulties adjusting to civilian life. |
| Waldhauser 2021 | Canada | Army and Navy | All male | After | Not stated | Honourably discharged | Not stated | participating in the sport-based programme |
| Westwood 2010 | Canada | Army | All male | Not stated | Not stated | Not stated | Not stated | Transitioning from military to civilian life |
| Williamson 2022 | UK | Not stated – RCT (70.7% Army) | Pilot: all male  RCT: 4.9% | After | Not stated | Not stated | Seeking formal mental health support | None |
| Wood 2023 | UK | No restrictions – Army 56.7%, RAF 27.3%, Navy 15.9%, more than one branch 6.8% | 100% | After | Not stated | Not stated | Mental health challenge in last 5 years | None |

##### **Supplementary File 5. Quantitative results for included studies**

| Study | Suicide | Other mental health | Addictions | Employment | Other |
| --- | --- | --- | --- | --- | --- |
| Alichniewicz 2025 | None. | None. | None. | None. | Results show a conversion rate of 28.5% (273/959) from the M-CARM survey to the Go-Beyond program. This rate is notably higher compared with similar internet-based self-help programs, such as VetChange (1033/22,087, 4.7%) and resources for gambling behavior (5652/8083, 14%), but lower than the MoodGYM program (82,159/194,840, 42.2%). Additionally, individuals were 1.64 (95% CI 1.17-2.28) more likely to enroll when they express a need in Purpose and Connection, and they were 1.50 (95% CI 1.06-2.18) times more likely to enroll when they express the need Beliefs About Civilians, compared with those without these needs. The overall completion rate for the program was 31% (85/273) and modules’ individual completion rates varied from 8.4% (17/203) to 20% (41/206). Feedback survey revealed high overall user satisfaction with Go-Beyond, emphasizing its engaging content and user-friendly modules. Notably, 94% (88/94) of survey respondents indicated they would recommend the program to other veterans, family, or friends. |
| Cox 2019 | None. | It was assessed whether the trimmed model (Model 2) better supported the social causation or social erosion model. From pre- to post-program, the social erosion model was supported: Pre-program PTSD attenuated pre-program to post-program changes in appraisal support (B = –0.157, p = 0.002) while there was no effect of any type of support on pre- to post-program changes in PTSD. Consistently, post-program PTSD attenuated changes in tangible support from post-program to 3-month follow-up (B = −0.133, p = 0.049) while there was no effect of any type of support on post- to follow-up changes in PTSD. Collectively, these findings support the social erosion model over the social causation model. | None. | None. | None. |
| Forces in Mind Trust 2021 | None. | In relation to mental health, use of the Short Warwick-Edinburgh Mental Wellbeing Scale (SWEMWBS) recorded a 23% uplift in clients’ mean SWEMWBS score for the statement ‘I’ve beendealing with problems well’, with uplifts for other statements ranging between 10% and 19%. | None. | None. | A net present value of the programme was calculated as £677,145.22 for the first year. The Social Return on Investment (SROI) value generated is expressed as a ratio of return of 4.74, indicating that for every £1 spent on delivering the Veterans' Advocacy People, £4.74 in social value is created. A sensitivity analysis has shown how extending the duration of outcomes by a single year, not an unreasonable assumption, (with drop-off set to zero) would see the social return value nearly double to £9.32 (this falls to £7.03 with a drop-off rate of 50% consistently applied). For now, this social return on investment analysis suggests that The Veterans' Advocacy People is  delivering considerable outcomes for the community it serves but also doing so in a cost-effective manner. |
| Gati 2013 | None. | None. | None. | In a pre- and post-workshop comparison, a statistically significant reduction of difficulties as measured by the CDDQ was found. The greatest reduction in difficulties was observed in the Lack of Information category. The workshop did not reduce difficulties involving Lack of Readiness or Unreliable Information. Participants reported actually having more Dysfunctional beliefs at the end of the workshop. Although it is unclear why this occurred, any conclusion drawn should be tentative, given the fact that the effect size was very small (d = 0.14). In addition, the self-reported severity of difficulties also significantly decreased, reflecting that the participants intuitively felt the objective decrease in career decision-making difficulties. While self-reported difficulties before participation in the workshop stemmed mainly from the Lack of Information cluster and the Inconsistent Information cluster, the self-reported difficulties after the workshop also stemmed from the Lack of Readiness cluster. Finally, although the participants were aware of the workshops' effect in reducing difficulties, the measured reduction (d = 0.57) was greater than that reflected in the subjective, self-reported difficulties (d = 0.36). This indicates awareness of the benefits of the workshop, but it also shows that measuring the difficulties objectively makes it possible to better evaluate where the benefit was greater (i.e., for difficulties involving Lack of Information) and where it was less (i.e., for Lack of Readiness).As hypothesized, there was a significant decrease in the total CDDQ score (t [1314] = 24.98, p < .001, d = 0.57), and especially in the Lack of Information difficulty cluster score (t [1314] = 29.99, p < .001, d = 0.78). The decrease was small in the Inconsistent Information cluster score (t [1311] = 10.57, p < .001, d = 0.25), and negligible in the Lack of Readiness cluster score (t [1314] = 3.96, p < .001, d = 0.09). Contrary to expectation, there was a slight increase in difficulties involving dysfunctional beliefs (t [1314] = −5.50, p < .001, d = 0.14). Finally, as hypothesized, the self-reported severity of difficulties also decreased to a moderate degree (t [1259] = 12.89, p < .001, d = 0.36). The correlation between the decrease in measured difficulties and the decrease in self-reported difficulties was .47, p < .001. | None. |
| Gillin 2021 | None. | None. | None. | 67% of spouses felt more confident in their ability to obtain work because of participating in the Trial, 78% stated a positive impact on their lives, and 80% would recommend it to others. | None. |
| Harper 2014 | None. | None. | None. | None. | All nine factors of the OBOI measured improved from pre-course to post-course (6 weeks later), these included self-confidence, goal setting, resilience, empowerment, problem solving, effective communication, group collaboration, compassion and envrionmental awareness. All produced medium to large effect sizes according to Cohen’s d. |
| Jones/ Quilgars 2014/2018 | None. | None. | None. | None. | Gaps in provision/areas for improvement. Adequacy of dedicated services for veterans. 36 veterans were waiting for 177 bed spaces across 13 direct access/ second stage schemes (across a number of regions of England and Scotland), and approximately 60 veterans were waiting for 157 units of long-term housing (in one scheme in London; in survey of providers' own experiences of adequacy. The  largest problem experienced was around social housing, in terms of long waits for access, an overall shortage and a shortage of suitable properties in the sector. Fewer providers reported  frequently experiencing problems in finding private sector housing than social rented housing, but also reported on the shortage of suitable private sector housing to the same  extent as for social housing. Fewer problems were reported in accessing housing advice and in finding housing in suitable locations). A majority (57%) of authorities assessed that there were sufficient and appropriate services  for veterans who were homeless or at risk of homelessness. There appeared to be a particular need for increases in independent accommodation,  particularly social housing (48% of authorities) and also private rented sector housing (37% of authorities). Overall authorities considered that they were supporting veterans in housing need quite (60%) or very well (12%), whilst a quarter (27%) of authorities were unsure of the extent to which they were meeting needs. Only 2% of authorities considered they were  poorly serving veterans in this area. Changing demand and provision in the future: dedicated providers felt that demand was likely to increase in almost all of the areas that they were currently working: in 16 areas, it was considered that demand  would increase significantly and, in nine areas, it was considered that demand would increase slightly. Authorities were less likely to predict an increase in demand in the next few years than providers, but were more likely to predict increases than decreases in veteran housing need. Dedicated veteran accommodation providers were asked to report on any development plans for new services in the near future. Twelve of the 17 providers reported that they had  undertaken needs assessment work that had identified the need for further service remodelling or developments for veterans in housing need. This information would suggest that dedicated provision for single veterans is likely to increase by at least 235 bed spaces/ units over the next few years. This would represent an  increase of 26% compared to the existing 910 bed spaces. At least a further 15 floating support places are also likely to be available. This would only represent a 5% increase on the present number of floating support places. it is likely that generic homelessness services will continue to reduce in scale given cuts in welfare budgets across Great Britain. |
| Metcalf 2022 | None. | Participants who received four sessions of attention-control training had significantly lower levels of PTSD symptoms and significantly improved functioning compared to those who received placebo attention training. Mean total PCL-5 scores, Hedges’ g = 0.86, 95% CI [0.37, 1.36], p = .004, and total WSAS scores, Hedges’ g = 0.93, 95% CI [0.46, 1.39], p = .001, at Time 2 were significantly lower among participants in the attention training group compared to the control group. The results showed that a significantly larger proportion of participants in the control group reported an increase of 5 or more points on the PCL-5 compared to the attention training group, 0.0%, χ2(1, N = 50) = 7.672, p = .006. | None. | None. | None. |
| Moran 2024 | None. | Linear mixed model results reveal statistically significant post-program reductions in symptoms of posttraumatic stress (PCL-M; *ß* = - 11.92, 95% CI [-17.44, - 6.36]), depression (MDI; *ß* = -7.87, 95% CI [-12.35, - 3.38]) and anxiety (GAD-7; *ß* = - 4.02, 95% CI [-6.42, - 1.57]), which were retained at 7-months follow-up. Clinically significant changes were also observed in all three outcomes. No changes were observed in the control group. Additional statistically significant beneficial effects for each model outcome were recorded for leisure surfing following the program. Most surf therapy participants continued surfing post programme, indicating effective lifestyle change. | None. | None. | None. |
| ONS 2024 | None. | None. | None. | None. | Key areas for service improvement: 46.7% training or additional training, learning new skills or a trade 37.1%, information or support services 37.0%, qualifications 31.7%, confirmed employment 32.9% - but among those who said they were prepared or very prepared for transition, the most popular response was none of the above 38.3%, but over 6 in 10 of these veterans still considered they would benefit from additional services. For those who felt to some extent prepared or to some extent underprepared, training or additional training (31.6%; 66.8%), learning new skills or a trade (23.7%; 54.4%); qualifications (22.6%; 44.1%); confirmed employment (22.4%; 46.8%); information or support services (21.6%; 57.8%). Older people were less likely to feel that additional services would be useful (80+: 40.2% none of the above vs 4.5% in 18-29). Females were more likely than males to have said, information or support services, mental health services, medical care or support, a family counsellor and a transition team or worker would have improved transition than male veterans. Conversely, they were less likely to have said qualifications (27.4% compared with 32.2%), alcohol support services (1.6% compared with 3.7%) and training or additional training (40.8% compared with 47.4%) would have improved transition than males. When considering types of information, advice or guidance veterans felt would have improved their transition to civilian life, veterans who were disabled were more likely to have selected each service proposed compared with those who were not disabled. The largest differences were seen for the proportions selecting medical care or support (20.5% compared with 6.9%) and mental health services (21.7% compared with 7.6%). As day-to-day activity becomes more limited, the proportion of veterans who said medical care or support would have improved transition increased (6.2% of those with no long-term physical or mental health conditions said medical care or support would have improved transition, compared with 29.1% of those whose day-to-day activity were limited a lot). The same patterns were seen for mental health services. Veterans who were medically discharged were also more likely to say all types of information, advice or guidance proposed would have improved their transition to civilian life than those who left the armed forces for any other reason. If a veteran had ever served as a regular (only as a regular or as a regular and reserve), they were more likely to select each of the proposed types of information, advice or guidance except for mental health services, alcohol support services and family counsellors where there were no differences by service type. Those who said they served in the British Army were much more likely to have selected confirmed housing as a service that would have improved transition to civilian life than those who said they served in the Royal Navy, Royal Marines or the Royal Air Force (25.1% compared with 14.0%, 14.2% and 16.0% respectively). Those who said they had served in the Royal Air Force were more likely to have said none of the types of information, advice or guidance suggested would have improved their transition than those who said they had served in other branches of the UK armed forces (26.4% compared with 18.3% of those who served in the Royal Marines, 21.7% of those who served in the British Army and 22.9% of those who served in the Royal Navy). Veterans who said they had served at Officer ranks were also more likely to say none of the options presented would have improved their transition to civilian life compared with veterans who said they served at lower ranks (36.5% compared with 18.2%). Veterans who witnessed or took part in operations against enemy forces were more likely to say all of the suggested types of information, advice or guidance would have improved transition to civilian life than those that did not. However, the largest percentage differences related to proportions selecting alcohol support services (4.3% compared with 1.8%) and mental health services (19.1% compared with 9.6%). Veterans who said they were subject to bullying during service were more likely to have said each of the suggested types of information, advice or guidance would have improved transition to civilian life than those who did not. One of the most notable differences related to the proportion who said mental health services would have improved transition to civilian life, the proportion of those who said they were subject to bullying and said this would have helped (24.7%) was over double the proportion of those who said they were not subject to bullying (9.9%). There were very similar findings when comparing veterans who said they were subject to discrimination during service with those that did not (29.4% compared with 11.2%) and when comparing veterans who said they were subject to harassment or sexual harassment during service with those who were not (29.5% compared with 12.8%). |
| Parkes 2023/2022 | None. | For the intervention group, symptoms of mental health distress (GHQ-12) decreased significantly (P<.001) from preintervention (mean 7.0, SD 3.6) to the 3-month follow-up (mean 2.7, SD 3.6) with a mean difference of 4.3 (95% CI 1.9-6.7). Symptoms of PTSD (PCL-C) decreased significantly (P=.03) from preintervention (mean 44.2, SD 15.0) to the 3-month follow-up (mean 33.2, SD 13.9) with a mean difference of 11.0 (95% CI 1.3-20.7). Well-being (WEMWBS) improved significantly (P=.04) from preintervention (mean 38.3, SD 9.2) to the 3-month follow-up (mean 44.9, SD 9.4) with a mean difference of −6.6 (95% CI −12.8 to −0.5). For the WHOQOL-BREF psychological subdomain, quality of life increased significantly (P=.01) from preintervention (mean 11.1, SD 2.6) to the 3-month follow-up (mean 13.3, SD 2.4) with a mean difference of −2.2 (95% CI −3.9 to −0.5). For the control group, there were no significant differences in any of the outcome measures from preintervention to the 3-month follow-up. | None. | None. | Technical delivery of the app was successful, with only minor resolvable technical issues identified by either the research team or the participants. |
| Romanuik 2024 | None. | Repeated measures ANOVA of participants’ scores on the Q-LES-Q-SF demonstrated no significant main effect, F(2, 42)=2.89, p=.06, ηp 2=0.121. Similarly, a series  of repeated measures ANOVAs were conducted to examine changes on participants’ scores on the DASS-21 and PCL-5. No significant main effect was found on DASS  subscales including Depression, F(2, 42)=2.55, p=.09, np 2=0.108, Anxiety, F(2, 42)=2.133, p=.13, ηp 2=0.092, and Stress F(2, 42)=0.386, p=.68, ηp 2=0.018 or on the  PCL-5, F(2, 42)=0.218, p=.81, ηp 2=0.010. | None. | None. | Planned comparisons of each timepoint using Bonferroni correction showed that there was a significant improvement in M-CARM scores from pre-intervention to postintervention (p=<0.001) and from pre-intervention to follow-up (p=<0.05). On a Likert scale of 1 to 5 (1=no improvement – 5=much improved), the average rating for improvement in adjustment was 3.6 and the average rating for improvement in reintegration was 3.4. Finally, when asked if they would recommend the program to other veterans, 100% of participants responded ‘yes’. |
| Westwood 2010 | None. | The results indicate improvement in trauma symptoms, depression and self-esteem. The average scores on the TSI were lower at posttest on all clinical scales except for an increase on the IE scale (Intrusive Experiences). Between the first and third admission, the rate of self-reported depressive symptoms and impression of self-esteem improved, self-esteem had no average change between the second and third administration while depressive symptoms increased slightly. A large effect was seen on the Tension Reduction Behavior (TRB) clinical scale. High levels of tension reducing behavior on the TSI suggest that participants felt less tension following the program. | None. | None. | None. |
| Williamson 2022 | None. | For the changes in quality of life domain scores, it was found that between baseline and day 84, as compared with to the control arm, the intervention arm showed significantly greater improvements in physical health (difference in score marginal means: 1.35 [95% CI=0.72 to 1.92]), psychological (difference in score marginal means=1.11 [95% CI=0.41 to 1.81]) and environment domains (difference in score marginal means=0.95 [95% CI=0.34 to 1.56] | RCT: participants in the intervention arm had significantly larger reductions in self-reported alcohol unit consumption from baseline (marginal unit mean=56.3 [95% CI=50.6 to 62.0) to day 84 (marginal unit mean=28.1 [95% CI= 21.1 to 35.1) compared with those in the control arm (from 54.0 [95% CI=48.2 to 59.8] to 43.5 [95% CI=36.3 to 50.8]; interaction p=0.006). between baseline and day 84, weekly alcohol consumption reduced by -10.5 [95% CI= -19.5 to -1.5] units in the control arm and -28.2 [95% CI= -36.9 to -19.5] units in the intervention arm (p-value for difference between arms at day 84=0.003). The difference in unit marginal means was -15.44 [95% CI= -25.52 to -5.35] units of alcohol in favour of the intervention arm. There was also evidence of a strong effect between the two groups by day 28, but no evidence of an effect between the two groups by day 168 for self-reported alcohol consumption. change in AUDIT score by day 84, we again found that participants in the intervention arm had significantly larger reductions in AUDIT score from baseline (score marginal mean: 16.3 [95% CI=15.0 to 17.5) to day 84 (score marginal mean=10.1 [95% CI=8.5 to 11.8) compared with those in the control arm (from 16.0 [95% CI=14.6 to 17.3] to 14.1 [95% CI=12.4 to 15.7]; interaction p=0.003). The difference was -3.9 [95% CI= -6.2 to -1.6] score on the AUDIT in favour of the intervention arm. There was no evidence of an effect between baseline and day 168 for AUDIT score. Participants in the intervention arm reported a median of 7 drinking days (IQR=4 to 11), a median of 3.5 drink free days (IQR=2 to 7) and a median of 12.8 units of alcohol per drinking day (IQR=4.4 to 16.5). | None. | Pilot: The Drinks: Ration app was rated as being easy to learn and use by expert users (mean=6.43; SD=1.13 and mean=6.14; SD=1.07, respectively) and veteran users (mean=6.43; SD=1.51 and mean=6.00; SD=1.41, respectively). Both expert (mean=6.57; SD=0.53) and veteran (mean=6.29; SD=1.25) users felt that the app adequately acknowledged and provided information that informed them of their progress. Expert users felt that the interface of the app allowed them to use all the functions (mean=6.71; SD=0.76) and they thought that the Drinks:Ration app provided an acceptable way to receive healthcare services (mean=6.71; SD=0.49). Veteran users felt the amount of time it took to use the app was fitting (mean=6.86; SD=0.38) and thought that the navigation was consistent when moving between screens (mean=6.57; SD=0.79). Veteran users did not agree that the app was helpful for their health and wellbeing (mean=4.00; SD=1.90) or that it helped them manage their health effectively (mean=4.33; SD=1.37). Generally, veteran users did not score Drinks:Ration highly for usefulness. Overall, participants were satisfied with the app (experts mean=6.43; SD=0.79 and veterans mean=6.00; SD=1.41). RCT: For the changes in quality of life domain scores, we found that between baseline and day 84, as compared with to the control arm, the intervention arm showed significantly greater improvements in physical health (difference in score marginal means: 1.35 [95% CI=0.72 to 1.92]), psychological (difference in score marginal means=1.11 [95% CI=0.41 to 1.81]) and environment domains (difference in score marginal means=0.95 [95% CI=0.34 to 1.56]. There was no evidence of an effect for social relationships between baseline and day 168. Over the study participants in the control arm used the app for a median of 1 week (IQR=1 to 2), initialised the app a median of 3 times (IQR=2 to 9) and had a median session duration of 60.9 seconds (IQR=35.7 to 75.6). Participants in the intervention arm used the app for a median of 3.5 weeks (IQR=2 to 6), initialised the app a median of 13.5 times (IQR=4 to 27) and had a median session duration of 43.8 seconds (IQR=32.3 to 67.9). A median of 18 push notifications (IQR=9 to 19) were sent to participants in the intervention arm, along with a median of 12 SMS text messages (IQR=10 to 14). Participants engaged with all modules of the app, but most of the app engagement was spent using the screening module (mean: 201.0 [SD=994.6) and normative feedback module (mean: 510.4 [SD=1012.7]). App usability questionnaire - participants respond to a set of usability questions on a scale of 1 to 7, with a higher value indicting improved usability. Participants in the control arm reported an overall app usability score of 4.1 (SD=1.5), ease-of-use score of 4.4 (SD=1.6), interface and satisfaction score of 4.1 (SD=1.6), usefulness score of 3.6 (SD=1.7). This was lower than the intervention arm, who reported an overall app usability score of 5.9 (SD=1.1), ease-of-use score of 5.9 (SD=1.2), interface and satisfaction score of 5.9 (SD=1.1), usefulness score of 5.7 (SD=1.1). In total, 2 out of 123 participants were identified as having a single adverse event of consuming more than 25 units of alcohol within 24 hours during the study period. There were no other adverse events identified. |

##### **Supplementary File 6. Qualitative results for included studies**

| Study | Suicide | Other mental health | Addictions | Employment | Other |
| --- | --- | --- | --- | --- | --- |
| Ackley 2023 | None. | None. | None. | Participants perceived the theatre-based programmes to have impacted them in multiple ways, through increased skills and confidence, therapeutically, driving their own purpose, social connections and trust, and employment opportunities. | Four themes were generated from this study: providing purpose, therapeutic storytelling, social connection, and the impact of the programme ending. Perhaps a key attribute to the success of the programmes is the ability to create a sense of purpose for participants. For some, the programmes provided a sense of purpose in terms of routine, skill development, and pride in participation. Like sense of purpose and routine, the social connections developed during the programme were reminiscent of their military social ties. This occurred for two reasons: firstly, their shared military experience and camaraderie, and secondly due to the close proximity and trusting setting of the theatre programme. |
| Albertson 2019 | None. | About their early engagement with the peer group recovery project, the narratives were suffused with enthusiasm and relief, describing a renewed sense of belonging to their veteran peer group. A communally shared, collective reflection of their shared past tapped into and reconnected with a communal structure and the military traits they both recognised and identified positively with. | None. | None. | None. |
| Bailey 2023 | None. | None. | None. | None. | Enablers for female veterans accessing services: collaborative, female-friendly services and support; high-quality services. Examples of best practice: health and well-being - co-creation of arts activities, comic books, delivered by women - range and availability of services differs across devolved areas of UK and can be difficult to access services in rural areas; challenge that female veterans do not often identify as veterans. Making a home - need to communicate available services more widely and more effectively, need more collaboration between military and non-military charities. Employment, education and skills - civilian employers and veterans do not fully appreciate the range of skills and knowledge veterans have, need to gain confidence in skills, need more collaboration between charities to make best use of available resources; need for support workers to understand the needs of female veterans. Community and relationships: female veterans often have difficulty identifying as veterans and as such consider services not relevant to them - one service shared how using a general community space without much about the military on the walls was beneficial; ensure that services are relevant to and do not place undue burden upon service users. Finance and debt: Many female veterans feel excluded and that the services on offer are not relevant for them. Financial issues are particularly sensitive for female veterans - it is important that female veterans feel charity workers are empathetic to their situation. Female veterans in the Criminal Justice System are very rare and have specific needs that need to be understood - importance of knowledge exchange and capacity building. |
| Belliveau 2019 | None. | The goal of the six interviewed participants in the play was focused on helping other veterans who might be suffering from posttraumatic stress injuries, showing successful approaches to coping with service-related trauma. However, engagement with artmaking and performing extended their own therapy, leading to significant personal changes for many of the performing veterans. | None. | None. | None. |
| Belliveau 2017 | None. | None. | None. | None. | The collective process was an expressive vehicle for military men to publicly reinterpret their struggles and experiences of transitioning from active service overseas to civilian life in ways that build resilience. The article offers insights on how research-based theatre as a methodological approach invites researchers to artistically present their understandings in a performative manner. |
| Brittain 2012 | None. | None. | None. | None. | Achievement (Importance of the military, Determination and focus, Setting goals is vital); Self-actualisation (Encouraging positive thinking and attitudes, Coping strategies, The importance of rehabilitation); Inspiration (The power of sport, The dedication of rehabilitation professionals, Enjoyment); Direction (Discovering and acknowledging limitations, Improvement in quality of life, Effective reintegration, Improved fitness, stamina and skills). Having applied the principles of life course theory to address the transition for military personnel who have endured life-changing trauma and are, through the use of sport, negotiating their post-injury status and place within society, an important finding in this research is the ways in which sport provides direction – helping individuals in their newly defined role and transition and re-integration into society. |
| Carins 2025 | None. | None. | They discussed the needs for veteran wellbeing programmes that are holistic and not focused on alcohol consumption in isolation. | None. | They spoke of need for opportunities for mental stimulation and development as well as 'challenge' to provide a meaningful post-service life. Physical wellbeing was recognised as important post-transition, examples included activities related to building and maintaining physical fitness like outdoor adventures, hunting, fishing. Social connection was described as a vital feature for programmes to support post-service life. They preferred programmes delivered in person rather than online. Spiritual wellbeing was recognised for veteran wellbeing, and they suggested employment or financial support in the early part of the transition. Participants emphasised importance of trust within veteran programmes, and veterans can be vulnerable during the transition. |
| Edwards 2022 | None. | None. | None. | None. | After intervention from Forward Assist and Salute Her UK, felt safe, not judged, listened to, for first time since being in military they know it's not their fault; learnt what choices you have in life; someone cares not just a number, feeling believed. Women involved in our research expressed a high level of understanding and insight into what could be done to help other women going through the service complaints process. Many wanted to help to re-design women friendly support services or train the Military Service Police, Assisting Personnel and Senior ranking officers on how best to deal with bullying, harassment, sexual assault and rape. |
| Forces in Mind Trust 2021 | None. | Health was identified as a key area of impact, with the  importance of positive outcomes in this area highlighted  by many, and uplifts in mental wellbeing documented.  The service was described as one which enables people to access the appropriate healthcare, and to avoid reaching  crisis point. | None. | A few clients went on to access education or secure a job, whilst others had their debt either cleared or made subject to a new and manageable payment agreement. Many were assisted in resolving housing issues and having aids/ adaptations carried out, some with accessing a mobility vehicle, and yet more received support in tackling financial and benefit issues. | Other positive changes included accessing new services (31 client cases); onward organisational engagements (51); improved health management (18); employment, training and education successes (11); social integration (5); and relationship support (7). Clients particularly highlighted the importance of having someone to speak on their behalf when they are having difficulties communicating. A key client outcome area was acknowledged around how The Veterans' Advocacy People enable help-seeking.  behaviour in clients who hitherto have struggled to acknowledge problems and seek out support. It helped them find their voice. Evidence shows that clients' confidence and resilience has been boosted by engaging with The Veterans' Advocacy People, another key outcome area. Others were on the brink of homelessness when  engaging with The Veterans' Advocacy People, managing to avoid that crisis point, something which delivers considerable wellbeing benefits. |
| Forces in Mind Trust 2013 |  |  |  |  | If there was one message that came from the stakeholder interviews and qualitative survey, it was this: the charities involved in the sector needed to collaborate more effectively and to make it easier for ex-Service personnel in search of help to find it. Qualitative research found that ex-Service personnel are aware that there are a significant number of organisations for people leaving the Services. However, most are unable to name the organisations, and even fewer are able to identify the specific needs or audiences they are intended to serve. Information about the organisations is often delivered as part of a long list of charities and web links; and there is limited support to navigate the list or find the relevant organisations. The qualitative research found a strong preference for resettlement information to be communicated and expectations managed one-to-one rather than through a formal group briefing. |
| Gillin 2021 | None. | None. | None. | Findings offered compelling evidence that employment support helped increase spouses’ confidence, provided new skills, and helped them to feel valued by the military. | None. |
| Godier-McBard 2021 | None. | None. | None. | Career Transition Partnership services were seen by several female veterans as being male orientated. Many recognised that flexibility was an important, yet under-catered for aspect of female veterans’ careers many of whom were leaving Service due  to caring responsibilities. | The improved targeting and signposting of veteran support services to women featured strongly in the stakeholder discussions and is found in the top three priorities for both stakeholder groups. Other common themes across the stakeholder groups included a better awareness amongst health and social care professionals of the needs of female veterans, and reducing the stigma and misconceptions caused by a lack of understanding/recognition of women’s experiences in the military. The importance of this issue in particular is reflected by its prominence in the top three priorities for each group. The remaining priorities reflect the majority of those made as a result of the scoping review and SME interviews. Broadly these appear to focus on the socio-economic needs of vulnerable groups of female veterans. Common themes across the groups included the need to identify gaps and barriers to support services, identifying the employment and financial needs of women, and reproductive health. Top priorities among female veterans were 1) Better signposting to appopriate support services 2) Use of female veterans as experts-by-experience in developing services 3) Development of an informal support network for UK female veterans 4) Better awareness and training in health and social care professionals to recognise and meet women's needs. Top priorities among Veteran support organisations were 1) Improve In-Service policy around culture of discrimination and harassment of minorities 2) Improve equality of opportunity for women in Service and female voices in senior roles. |
| Harper 2014 | None. | None. | None. | None. | Four major themes included 1) Same but different 2) Decompression/awareness raising 3) Healthy community and 4) Stigma free. Participants described the training environment of OBC as parallel to CF training (skills training, camaraderie, physicality etc). Most left the course with an increased understanding of their common 'soldier's experience' and potential subsequent health issues. Finding meaning through intensive group dialogue or physical activity provdied some participants with ignition to work on issues arrising for them. Participants acknowledged the value of 'rank' not being disclosed to ensure equality and increased cohesion. Many recognised that the course lessened the effects of stigma, but acknowledged hurdles remain. |
| Healthwatch Hertfordshire 2021 | None. | None. | None. | None. | Veterans noted that additional information about the healthcare services available to veterans would have been helpful when transitioning into civilian life and would have encouraged them to access this support. Veterans also highlighted that most GP practices are not aware of the services and support available to veterans, and do not offer specific signposting information or referrals to services exclusively for veterans. Veterans noted that they prefer to seek help from charities and local veteran support groups, as they have a much better understanding of their needs. The findings showed that most GP practices do not record or identify veterans, meaning there is an onus on veterans to disclose their military service. However, it is evident that veterans are often reluctant or not aware of the importance of sharing their military history with NHS services. It is important veterans are identified and recorded by GP practices, as flagging veterans means they can better support them, by enabling access to priority treatment and signposting and referring veterans to the appropriate care. |
| Heaver 2018 | None. | Much greater attention needs to be paid to the ‘softer’, less practical elements of transition with support for service leavers and family members to help them prepare for what they might experience during and after transition with regards to the emotional and psychological effects that can be associated with such a significant life change. Consideration needs to be given to the emotional wellbeing and resilience of families during transition and how best they can prepare for, and seek advice where necessary for what they, and the service leaver might experience during transition. | None. | None. | Families indicated that they did want to be involved in their Service leaver’s (SL) transition. They demonstrated that they too were affected by transition. New transition support should be developed specifically for families. For families to be fully and positively engaged in the transition process, information needs to be made directly available to them. Consideration needs to be given to ensuring information is presented in a way that is physically accessible via well-marketed websites supported by social media, and that the language, tone and style of transition information is more relevant and understandable. Families need help with understanding the financial realities of transition, especially the costs involved with living outside of the Services.Attention needs to be paid to the ‘softer’ elements of transition and the value placed by families on informal, non-Service led support networks, such as friends and neighbours. How can these channels be utilised to best effect? The MOD should invest in an education piece across the Services to raise families’ awareness of the inevitability of transition and the importance of early preparation. This needs to be done in a suitably engaging way, directly to families. |
| Hooks 2023 | None. | None. | None. | None. | Facilitators to accessing support services for female veterans included service providers who have military knowledge and understanding. Peer support from other veterans, especially those who have had positive experiences with military or veteran support services, also acted as a significant facilitator to accessing support. Female-specific initiatives and service providers were seen as valuable for creating a ‘safe space’ where female veterans could discuss gender-specific issues and feel more comfortable. Positive previous experiences with veteran-specific services and the sense of belonging and camaraderie provided by these services also facilitated access to support. Regarding the need for female-specific services, participants’ opinions varied. Some felt that there was no need for female-only services and identified more with their military role, emphasising the importance of connecting with services as veterans rather than as women. However, participants who saw a need for female-specific services argued that existing services were designed for and by men and did not address the unique needs and experiences of female veterans. Participants highlighted the need for healthcare professionals to be aware of female  veteran needs in gendered areas of care, such as the perinatal period, menopause, and  infertility. |
| Hutcheon 2024 | None. | None. | None. | The employment training programme was found to impact veterans by providing validation and formal recognition of the skills and knowledge they already had, by preparing them for employment through learning new skills, and developing or refreshing existing skills, raising their awareness of the need for formal training, and allowing them to demonstrate a continued willingness to learn new things. Most participants reported that they got confidence from learning new skills and gaining new knowledge from the progressive employment training programme. In learning new skills, and also refreshing their knowledge, two participants stated that they had a greater sense of pride in themselves and self-worth. Gaining accredited qualifications gave participants a greater sense of confidence in being work-ready or more employable. | A large proportion of the participants emphasised that the success of the delivery of the programme was down to the support and encouragement of the Training Manager and their understanding of the needs and capabilities of each veteran. |
| Mackintosh 2025 | None. | None. | None. | None. | Challenge and competitive sports played a pivotal role in their mental and physical recovery. As participants trained towards significant challenges, they utilised sport and PA as a form of self-medication. For many, this was a way of feeling accepted and perceived as  “normal”, even if it meant utilising adapted forms of activity. |
| Meierdirk 2024 | None. | None. | None. | The support received for resettlement has changed drastically over the last 10 years. Several veterans posited how much better the support was now than 20 years ago. Three of the veterans were positive about their experience with the programme and although they did suggest areas for improvement, they were happy with their experiences. Many of the veterans did use their allowances to attend courses such as Participant 8: “I actually did a locksmith course which was partially funded through the enhanced learning credits, which is £2000”.One of the biggest complaints about the CTP was the support received varied between the services and locations; it lacked consistency and many of the participants discussed this variability. The veterans believed the support needed during the transition varied due to numerous factors. Age was the main factor quoted, unfortunately, the support given was not adjusted for this variable. There is a difference between the support a young 20-year-old needs, after 5 years of service, compared to service personnel who has served 20 years and is leaving with years of experience and a pension. Another area of improvement commented on was better signposting for leavers. Most of the veterans were very positive about the course, contents and teaching. Many of the service personnel benefited from help writing a curriculum vitae (CV), specifically for the construction industry and “linking their skills” to construction jobs. An area for improvement, which was also cited by the veterans, was they would have benefitted from more assistance setting up as self-employed in the construction industry. | None. |
| O'Connor 2017 | None. | Feedback suggests that the theatrical intervention Coming Home has significant potential as a way of reconnecting veterans and families with their community and improve their wellbeing. The emotional release of singing and performing together was considered powerful. Participants considered that the programme connects them with parts of themselves that they thought had gone forever: humour, spontaneity, fun - and having a positive impact on their wellbeing. | None. | None. | None. |
| O’Connor 2015 | None. | None. | None. | None. | Impact - not therapy but definitely therapeutic, follow-up interviews - project had given temporary increase in well-being and self-esteem, social connections, sense of belonging, pride on stage, sense of achievement, useful approach that enabled group cohesion, provided a structure for painful experiences to be shared and heard and was instrumental in shaping the story that the group presented to an audience. |
| O’Hanlon 2024 | None. | None. | None. | None. | Neoliberal systems foster an atmosphere of frustration, fear, paranoia, and competition, dimini  sh a sense of belonging, and encourage a fixation on being able to demonstrate individual achievements so they are appropriately rewarded - the need for staff to demonstrate their work, effort and success was felt within the AP and ultimately diverted attention away from core work objectives - the central aims of VFP addressing mental health, addiction and social isolation, are complex issues tied to contextual factors and broader dynamic processes and as such difficult to quantify for staff targets. Looking beyond quantifiable and measurable results to place value on data gained via participatory research methods, ethnographies, narrative analysis and qualitative methods. These approaches may produce video, storytelling and community-led outputs, that offer rich and complex data, through which the processes occurring with programmes can be unpicked. |
| ONS 2024 | None. | None. | None. | None. | Themes for what services would benefit (qualitative free text): None 33.2% - subtheme 22% of these 'too late now' but could have done with help. Health (just over 20% - various topics) - mental health, NHS, dental services, lack of services for injuries, health issues due to service, hearing loss, disability, War Pension, continuity of care (they add % quant data to the qual method but the qual is the essence here). Signposting and coordination (8.2%) - knowing what's available, a need for less fragmentation + improved communication. Careers services (5.7%). Housing (5.6%). Finance - often mentioned alongside housing, education, disability or pensions (5.2%). Pension (4.5%). Need for veteran identification (4.7%). Cultural and more general transition (2.8%) - wider understanding promotion and enforcement of Armed Forces Covenant and Duty of Due Regard, check in opportunities for veterans, networking opportunities, continued connection to armed forces, local services, recognition of veterans. These results were broadly qualitative, although some descriptive statistics were added. |
| Romanuik 2024 | None. | None. | None. | None. | Regarding the most beneficial part of the program as perceived by participants. Common ideas included ‘learning about self’ (Learning to deal with my triggers and with being ex-military and living in today’s society), ‘being in a group’ (Being with and meeting other veterans with issues relevant to myself), and ‘tools and information’ (Tools to manage symptoms). |
| Waldhauser 2021 | None. | Participants report a variety of positive benefits (mental and physical health, social connections, and access to resources), as well as military identity as a means of supporting social connectivity. The participants also commented on salient environmental features of the program that supported their involvement, as well as suggestions for program refinement. The study provides evidence for the feasibility and acceptability of the PASS program as well as insights into veterans’ experiences of this initiative. | None. | None. | None. |
| Westwood 2010 | Two participants who had been suicidal prior to the group reported more investment in life. | It is evident that the qualitative results support the quantitative findings of improved participant well-being upon completion of the program. Participants identified being with other military personnel as the single most important factor for their experience of change during the VTP. Participants perceptions of the facilitators as being warm, caring people who showed openness and genuineness was the second most helpful factor. Participants reported reductions in symptoms of PTSD and depression, and less vigilance, more optimism, hopeful feelings and enhanced wellbeing and life enjoyment. Two participants identified increased sleep quality. | None. | None. | Eight reported gaining self-respect and letting go of feelings of shame and guilt. Six reported improved emotional and behaviour regulation. The participants reported improved relationships with others, six felt an increased sense of trust with others. |
| Williamson 2022 | None. | None. | None. | None. | Pilot study: Overall, positive participant feedback suggested that the Drinks: Ration app had high usability for the target population of help-seeking veterans. Participants also provided several recommendations to further improve the usability of the app. Although the most used feature of the app was the drinks diary, participants also reported the goal setting and drinking recap to be important and beneficial for the target population. Initially, the two participant groups were analysed separately but are reported together due to the converging of themes across the groups. Three overarching themes (with sub-themes) – Simplicity (suitable for a range of technical abilities; study enrolment; easy to use and navigate); Appropriateness for Veterans (non-judgemental language; targets underestimation of alcohol consumption; utility for wider military community; ‘how you compare’ – the value of comparing to other veterans; relevance and consolidation); and Engagement (comparison to other apps; notification content and frequency; personalisation; informed by drinking severity). |
| Wood 2023 | None. | Veteran-specific mental health services should be responsive to the gendered nature of care needs and the care preferences of women veterans. Veteran-specific services should adapt their branding to ensure visibility of women veterans in imagery and to make it clear that services are not just for combat-related trauma. Improvement needs to be made to the understanding and training amongst all NHS staff with regards to women veterans’ mental healthcare needs and to raise awareness of NHS veteran-specific services and the support they can provide for women. | None. | None. | None. |

##### **Supplementary File 7. List of included records**

1. Ackley J, Wilson-Menzfield G. The theatre as therapy for military veterans? Exploring the mechanisms which impact psychosocial well-being and social connections during theatre-based programmes. Arts Health 2023; 15(1): 53-70
2. Albertson K. Relational legacies impacting on veteran transition from military to civilian life: trajectories of acquisition, loss, and reformulation of a sense of belonging.
   Illn Crises Loss 2019; 27(4): 255-73.
3. Alichniewicz KK, Hampton S, Romaniuk M, Bennett D, Guindalini C. Use of Go-Beyond as a Self-Directed Internet-Based Program Supporting Veterans’ Transition to Civilian Life: Preliminary Usability Study. JMIR Formative Research. 2025;9.
4. Bailey M, Phillips, R. and Morrison, Z. Improving access to service charities for female veterans.; 2023.
5. Belliveau G, Cook C, McLean B, et al. Thawing out: Therapy through theatre with Canadian military veterans. Arts Psychother 2019; 62: 45-51.
6. Belliveau G. Theatre with veterans: Transition and recovery. J Appl Arts Health 2017; 8(2): 255-65.
7. Brittain I, Green S. Disability sport is going back to its roots: rehabilitation of military personnel receiving sudden traumatic disabilities in the twenty-first century. Qual Res Sport Exerc Health 2012; 4(2): 244-64.
8. Carins J, Kennedy AM, Veer E. Co-Design of an Ecosystem of Services to Support Veteran Well-Being and Reduce Excessive Alcohol Consumption. Health Expectations. 2025;28(2).
9. Cox DW, Baugh LM, McCloskey KD, et al. Social causation or social erosion? Evaluating the association between social support and PTSD among Veterans in a transition program. J Mil Veteran Fam Health 2019; 5(1): 71-9.
10. Edwards P, Wright T. No Man's Land 1. 2019.
11. Edwards P, Wright T, Honor S. No Man's Land 2. 2022.
12. Forces in Mind Trust. The veterans' advocacy people: final evaluation report and social return on investment analysis. 2021.
13. Gati I, Ryzhik R, Vertsberger D. Preparing young veterans for civilian life: The effects of a workshop on career decision-making difficulties and self-efficacy. J Vocat Behav 2013; 83: 373-85.
14. Gillin N, McShane T, Gribble R, et al. UK veterans family study: Understanding and mapping the psychosocial wellbeing support needs of veteran family members across the UK: a multi-methods study. UK veterans family study; undated.
15. Godier-McBard L, Gillin,N., Fossey, M. We Also Served: The Health and Well-Being of Female Veterans in the UK. Veterans and Families Institute for Military Social Research, Anglia Ruskin University; 2021.
16. Harper NJ, Norris J, D’astous M. Veterans and the outward-bound experience: an evaluation of impact and meaning. Ecopsychology 2014; 6(3): 165-73.
17. Healthwatch Hertfordshire. Improving healthcare access for veterans. 2021.
18. Heaver L, McCullough K, Briggs L. Lifting the Lid on Transition: The families' experience and the support they need. Forces in Mind Trust. 2018.
19. Hooks C, Morgan, L., Fossey, M., Buxton E., Godier-McBard, L. ‘Where are all the women?’ Recognition and representation – UK female veterans’ experiences of support in civilian life.; 2023.
20. Hutcheon D, McMillan C, Morrison-Clancy S, et al. Accredited vocational training within supported employment for veterans: An evaluation of impact within Scotland’s Bravest Manufacturing Company. Glasgow Caledonian University; 2024.
21. Jones A, Quilgars, D., O’Malley, L., Rhodes, D., Bevan, M., Pleace, N.,. Meeting the housing and support needs of single veterans in Great Britain. . University of York.; 2014.
22. Mackintosh C, Reavill N, O’Hanlon R, et al. Understanding the role of invictus games in supporting the transition for UK disabled military veterans. Front Sports Act Living 2025; 7:1609967.
23. Meierdirk C. Building Heroes: building bridges to support the transition from military to civilian employment in the construction industry. Educ Train 2024; 66 (2-3): 166-80.
24. Metcalf O, O’Donnell ML, Forbes D, et al. Attention-control training as an early intervention for veterans leaving the military: A pilot randomized controlled trial. J Trauma Stress 2022; 35:1291-9.
25. Moran R, Sperandei S, Peel N, et al. The long-term effect of surf therapy on posttraumatic stress, depression, and anxiety symptomology among current and former Australian defence force members - A nonrandomised controlled longitudinal study in a community setting. Ment Health Phys Act 2024; 27: 100629.
26. O’Connor A. Coming home to the arts: theatre with military veterans and families. J Public Ment Health 2017; 16(1): 12-4.
27. O’Connor A. Abandoned Brothers, life story theatre with veterans and their families. Arts Health 2015; 7(2): 151-60.
28. O’Hanlon R, Mackintosh C, Thorpe H. Navigating Neoliberalism: The Realities and Challenges of a Veteran Sport Programme in the UK. J Sport Dev 2024; 12(2): 76-91.
29. Office for National Statistics. Preparedness to leave the UK armed forces: Veterans’ Survey 2022, UK. 2024. Available from: <https://www.gov.uk/government/publications/preparedness-to-leave-the-uk-armed-forces-veterans-survey-2022-uk/preparedness-to-leave-the-uk-armed-forces-veterans-survey-2022-uk>
30. Parkes S, Croak B, Brooks SK, Stevelink SAM, Fear NT, Rafferty L. MeT4VeT: development and assessment of a mental health toolkit for military veterans. Forces in Mind Trust. 2022.
31. Parkes S, Croak B, Brooks SK, Stevelink SAM, Leightley D, Fear NT, et al. Evaluating a Smartphone App (MeT4VeT) to Support the Mental Health of UK Armed Forces Veterans: Feasibility Randomized Controlled Trial. JMIR Mental Health. 2023;10:e46508.
32. Romaniuk M, Saunders-Dow E, Brown K, et al. Feasibility, acceptability, and initial outcomes of a psychological adjustment and reintegration program for transitioned military veterans. BMJ Psychology 2024; 12: 597.
33. Waldhauser KJ, O’Rourke JJ, Jackson B, et al. Purpose after service through sport: a social identity-informed program to support military veteran well-being. Sport Exerc Perform Psychol 2021; 10(3): 423-37.
34. Westwood MJ, McLean H, Cave D, et al. Coming home: a group-based approach for assisting military veterans in transition. J Spec Group Work 2010; 35(1): 44-68.
35. Williamson C, Simms A, Shearer J, et al. Drinks:Ration The role of a smartphone application in reducing alcohol consumption in a veteran population seeking formal mental health support. Forces in Mind Trust; 2022.
36. Wood A, Fossey M, Price P, Powell D, Chalkley H, Davidson M, et al. I don’t feel like that’s for me. Overcoming barriers to mental healthcare for women veterans. Centre for Military Women's Research.; Undated

##### **Supplementary File 8. Intervention details from included studies**

| Study | Intervention details |
| --- | --- |
| *Arts-based* |  |
| Ackley 2023 | This study focuses on theatre-based programmes run by a national charity in the UK aimed specifically at helping military veterans’ recovery through the arts. Professional theatre writers and directors were hired to produce the standards needed for a professional production, and to make sure the veterans’ stories were replicated truthfully. Professional actors and dramaturgs were used for the first part of the programme to develop acting skills, and professional counsellors were on hand if any participants exhibited psychological distress. The programmes were set up to improve the confidence and motivation of military veterans by supporting rehabilitation and aid their transition into civilian life, as well as improving the overall emotional well-being of the participants. |
| Belliveau 2017 | The Contact!Unload theatrical intervention uses theatre and visual art to engage veterans to share stories of trauma and pathways towards recovery. A series of drama-based workshops were run for three months, involving community members, artists, researchers, and veterans. The theatre project stems from Therapeutic Enactment, a group counselling strategy that involves enacting the narrative of critical events, going beyond language to express the self through action, movement, emotion, and reflection. |
| Belliveau 2019 | Contact!Unload theatrical intervention |
| O’Connor 2015 | Re-Live is a UK-based organisation working in the field of Arts and Wellbeing. The company creates professional theatre productions with non-actors who tell their own stories onstage. This practice-based report explores the process and impact of their 3-month theatre project with military veterans who experienced conflict. |
| O’Connor 2017 | Coming Home is a theatre programme from the UK-based Re-Live organisation. A group of eight veterans and five family members worked with Re-Live practitioners and a guest practitioner to explore experiences of coming home. |
| *Sports or outdoor based* |  |
| Brittain 2012 | This paper investigates the forged links between the military and the National Paralympic Committees in Australia, Canada, Great Britain and the USA. Five known military-paralympic programmes include Wounded Warriers, Soldier on, Paralympic military program, Battle back, and the Australian defence force Paralympic sport program. |
| Harper 2014 | Outward Bound Canada run adventure-based resiliency training courses in wilderness settings for both serving and retired personnel. Outward Bound Canada veterans’ courses use a standardized format composed of a 7-day wilderness expedition with up to nine participants and three instructors. The courses utilize an intensive group setting which enables peer mentoring and the sharing of deployment and post deployment issues in a structured and physically and emotionally safe environment. There is no rank on course. Course activities include hiking and backpacking, rock and ice climbing, ski mountaineering, alpine climbing, and white-water canoeing, along with a broad range of experiential activities commonly used by OBC to support positive personal and group development outcomes. Courses are designed to purposely mirror and reframe selected military experiences and offer opportunities for participants to reflect on and discuss their deployment experiences and transition challenges and the impact these have on their lives. |
| Mackintosh 2025 | The Invictus Games use sport and physical activity as a tool for rehabilitation and aim to foster understanding and respect for the military community. This report focuses on the experiences of veterans recruited via the Wounded, Injured and Sick (WIS) Invictus Games Foundation page. |
| Moran 2024 | Surf therapy sessions were held twice a week in Australia, for four hours. Each session consisted of individual goal setting, surfing practice, and informal social interaction over lunch and coffee. To reduce attendance barriers (such as personal/work schedules, or illness fluctuations) a rolling cohort format was used, whereby participants were allowed to commence at any time during the intervention period, but asked to attend once per week. They were allowed to complete any missed sessions on an alternate session or by extending the program period. |
| O’Hanlon 2024 | This paper examines the design, development and delivery of the Sport for Development (SFD) programme in the United Kingdom. The SFD is defined as the intentional use of sport, physical activity and play to attain specific development objectives. |
| Waldhauser 2021 | Purpose After Service through Sport (PASS) is a sport-based program in Canada that aims to complement existing veterans’ services. PASS is open to all veterans, not just those injured or ill. It invites veterans and current-servicing military to attend drop-in “ball hockey” on Friday evenings with a view to: (a) improve your health and physical fitness, (b) network and connect for military and civilian needs, (c) blow off some steam in a semi-competitive environment, and(d) connect and learn about support services and opportunities for former and leaving personnel. Ball hockey games lasted approximately 45–60 minutes and were followed by opportunities to socially connect afterwards. |
| *Group-based* |  |
| Albertson 2019 | This was a community-based veteran-specific peer group recovery project that focused on belonging and access to a secure place. |
| Cox 2019 | Canadian veterans enrolled in a multimodal outpatient group transition program. The program was designed to address emotional, interpersonal, and career-related barriers to transition using primarily cognitive-behavioural and group-process theory. The program took place in retreat centres over 10 eight-hour days. This retreat-style format was used to offer convenience over traditional weekly sessions and aimed to facilitate a cohesive context similar to the military experience. The program was divided into three phases, the first focused on group cohesion, psychoeducation about PTSD, emotion regulation skills training, and identifying important life events. Phase 2 involved enacting traumatic events within a group to modify maladaptive narratives, while phase 3 focused on identifying personal, relational, and career goals. |
| Gati 2013 | Young veterans took in a workshop held on five consecutive days for seven hours a day. The workshop took place in a community centre near the participants' neighborhood. The workshop aimed to (a) prepare the veterans for coping with changes in life-style, (b) enhance their self-esteem, (c) increase decision-making self-efficacy in general and career decision-making in particular, (d) set personal goals and suggest ways of actualizing these,(e) increase awareness of the importance of higher education, and (f) facilitate self-exploration. The second day focused specifically on career decision making. The other days involved outdoor training that simulated real-life situations in the world of work, including a visit to a nearby college, discussions of budgeting, employees' rights, interpersonal communication, as well as resume writing and preparation for job interviews and evaluation centres. At the end of the workshop each participant created an individualised plan for transitioning into civilian life. |
| Romanuik 2024 | Veterans completed eight weekly 2.5-hour group sessions designed to support transition to civilian life. The overall aim was to highlight psychological and cultural aspects that might hinder adjustment to civilian life. The program involved cognitive-behavioural and acceptance and commitment therapy techniques, with the aim of helping participants to identify and utilise new knowledge, skills, and strategies to facilitate improved adjustment. The program involved validation of past experiences while focusing on current needs and opportunities for healthy adjustment through individual reflections, group discussions and personal goal setting. The program covers eight sessions including; Social Connections, Communication Skills in Civilian Life, Finding Purpose, Identity, Beliefs About Civilians, Resentment and Regret, Regimentation, and Help-Seeking. |
| Westwood 2010 | The Veterans Transition Program (VTP) uses a residential group approach to support veterans in their transition and integration into Canadian society. The programme focuses on: (a) creating a safe, cohesive environment wherein soldiers can experience mutual support; (b) normalising of the soldiers’ military experiences overseas and the difficulties with re-entry back to civilian life; (c) offering critical knowledge to understand trauma and its origins and the impact on the self and others, as well as self-regulation strategies; (d) reducing the symptoms of the stress-related issues arising from their military experiences; (e) teaching of interpersonal communication skills to help manage difficult interactions or enhance relationships with others; (f) generating life goals and learning how to initiate career exploration; and (g) involving spouses and other family members in family awareness sessions. The format of the program is several weekend sessions. Activities involve writing short autobiographical accounts on pre-selected themes and therapeutic enactment. |
| *Employment/skills-based* |  |
| Hutcheon 2024 | Over the course of 12 or 18 months, veterans employed at Scotlands Bravest Manufacturing Company (SBMC) took part in a progressive employment training programme aimed at supporting veterans who were struggling to successfully find employment, or find employment that was suitable to their needs. Individuals on the progressive employment training programme were employed on a living wage gaining both specific vocational skills and generic skills relating to employability. Components of the progressive employment training programme included training needs welfare and physical needs assessment, analysis and personal development plan, on the job training and continuous assessment and self-reflection, and employability training. As part of the progressive employment training programme, a Training Manager was employed to design, develop and implement a range of training and qualifications for veterans employed at SBMC. Some of the accredited qualifications offered to veterans included NVQ 2 Performing Manufacturing Operations, Health and Safety in the Workplace, Fire Safety, Numeracy, and Workplace Core Skills. |
| Meierdirk 2024 | The Building Heroes course provides construction industry training for military service personnel transitioning into civilian employment. The course includes level 1 construction, level 2 welding and the operation of plant machinery. These courses are taught in partnership with further education and higher education providers and can last up to 5 weeks. |
| Metcalf 2022 | This was a pilot randomised controlled trial of attention-control training for personnel transitioning out of the military in real-world clinical service. Attention-control training was a variation of the dot-probe task, and involved four sessions, each lasting 10 minutes, delivered over 4 weeks. Veterans were presented with both neutral (e.g., “chair”) and threat-related (e.g., “death”) words and asked to respond to a target cue, per instructions issued at the beginning of the task. |
| *Digital* |  |
| Alichniewicz 2025 | “Go-Beyond, Navigating Life Beyond Service” is an evidence-informed, self-directed, web-based program designed to support veterans’ adjustment to civilian life. The program aims to (1) provide education about topics that are important for psychological adjustment and reintegration to civilian life; (2) build psychological insight into personal challenges adjusting to civilian life; (3) offer practical first steps and tools to facilitate stronger adjustment and reintegration; and (4) provide links to additional services and support available in Australia. The content of the program is based on previous research describing empirically derived psychological and cultural factors associated with adjustment and reintegration to civilian life, including (1) Purpose and Connection, (2) Help Seeking, (3) Beliefs About Civilians, (4) Resentment and Regret, and (5) Regimentation. These factors were derived as part of the development of the Military-Civilian Adjustment and Reintegration Measure (M-CARM). When accessing the Go-Beyond website, veterans are asked to complete this measure, which produces a unique M-CARM profile of results specifying potential areas of need on the 5 factors of the measure. |
| Parkes 2022/2023 | MeT4VeT: Development and Assessment of a Mental Health Toolkit for Military Veterans. This app was designed to help veterans in identifying when their experiences indicated a mental health problem, and to recognise when to seek help before reaching a crisis point. It was also intended to inform users of ways in self-manage symptoms and seek formal support. The app consists of five main sections designed to represent the intersection between core components of cognitive behavioural therapy (CBT) and behaviour change. These include People, Tasks, Tools, Tracking and Notifications. Signposting information is always available, and users are prompted to look at the signposting information if they input worsening mental health two weeks in a row. |
| Williamson 2022 | Drinks:Ration is an app designed to support veterans drinking at a hazardous or harmful level by providing bespoke advice and support over a 28-day period. The app was designed to enhance user motivation and self-efficacy in modifying their alcohol consumption using behahviour change techniques embedded in the content and messaging. The app uses interactive features throughout and includes five core modules: Account management, Questionnaires and individualised feedback, Self-monitoring and feedback, Goal setting and review, and Personalised messaging. |
| *Co-designed workshop* |  |
| Carins 2025 | This was co-designed workshop aimed at reducing problematic alcohol use among veterans transitioning. The study involved four workshops with veterans and health professionals in New Zealand, who made recommendations for a co-designed veteran well-being ecosystem. |
| *Individual charities or organisations* |  |
| Edwards 2022 | This study reviewed the support services available via the Salute Her UK. |
| Forces in Mind 2021 | This report reflects an independent three-year evaluation of The Veterans' Advocacy People. The Veterans' Advocacy People service is targeted at veterans and their families from each of the service arms. It aims to provide open and flexible advocacy support, assisting them to navigate the myriad of services available, and ultimately empowering them to find solutions and deal with the issues they face. |
| *Multiple service evaluations* |  |
| Bailey 2023 | Multiple service charities for female veterans were evaluated |
| Forces in Mind 2013 | This report from the Forces in Mind Trust (FiMT) set to understand how the transition process currently works and how it is viewed by stakeholders and recent service leavers, to develop a model that quantified the costs of poor transition to the UK. The report makes recommendations on how to reduce the number of poor transitions. |
| Gillin 2021 | Interviews were conducted with 11 service provider organisations and with 71 family members (20 England, 15 Scotland, 20 Wales, 16 Northern Ireland) |
| Godier-McBard 2021 | Almost thirty organisations, drawn from across the military charity sector as well as from statutory bodies including NHS England and NHS Improvement and the MOD contributed to this report on the health and wellbeing of female veterans. |
| Healthwatch Hertfordshire | The Hertfordshire Armed Forces Covenant Board, known locally as “Hertfordshire Heroes”, investigated the healthcare needs and experiences of veterans in Hertfordshire |
| Heaver 2018 | Over 70 information, advice or support services were reviewed relating to family transition. |
| Hooks 2023 | The Centre for Military Women’s Research (CMWR) was launched by the Veterans & Families Institute for Military Social Research (VFI). The CMWR seeks to inform and improve the well-being of women in the military and veteran community by co-production and collaboration in research and evaluation. This report aims to provide an in-depth understanding of the lived experiences of UK female veterans in relation to government and charity provision. |
| Jones / Quilgars 2014/2018 | These studies evaluated the relative effectiveness of dedicated supported housing services across the UK. |
| ONS, 2024 | The Veterans’ Survey 2022 aimed to capture preparedness to leave the UK armed forces. |
| Wood 2023 | The Veterans & Families Institute for Military Social Research (VFI) conducted research into the barriers to mental healthcare among women veterans |
